# Formation and ligand-based reductive chemistry of bridged bis-alkylidene scandium(iii) complexes†
†Electronic supplementary information (ESI) available. CCDC 1555495, 1504242, 1519012 and 1539530. For ESI and crystallographic data in CIF or other electronic format see DOI: 10.1039/c7sc02018j



**DOI:** 10.1039/c7sc02018j

**Published:** 2017-07-13

**Authors:** Wangyang Ma, Chao Yu, Yue Chi, Tianyang Chen, Lianjun Wang, Jianhao Yin, Baosheng Wei, Ling Xu, Wen-Xiong Zhang, Zhenfeng Xi

**Affiliations:** a Beijing National Laboratory for Molecular Sciences , MOE Key Laboratory of Bioorganic Chemistry and Molecular Engineering , College of Chemistry , Peking University , Beijing 100871 , China . Email: wx_zhang@pku.edu.cn; b State Key Laboratory of Elemento-Organic Chemistry , Nankai University , Tianjin , 300071 , China; c School of Chemistry and Chemical Engineering , Hunan Institute of Engineering , Xiangtan , 411104 , China

## Abstract

The chemistry of rare-earth carbene and alkylidene complexes including their synthesis, structure and reaction is a challenging issue because of their high reactivity (or instability) and the lack of synthetic methods. In this work, we report the first synthesis of the bridged bis-alkylidene complexes which feature a 2-butene-1,1,4,4-tetraanion and four Sc–C(sp^3^) bonds by the reaction of 1,4-dilithio-1,3-butadienes with ScCl_3_. This reaction proceeds *via* two key intermediates: an isolable scandacyclopentadiene and a proposed scandacyclopropene. The scandacyclopentadiene undergoes β,β′-C–C bond cleavage to generate the scandacyclopropene, which then dimerizes to afford the bridged bis-alkylidene complex *via* a cooperative double metathesis reaction. Reaction chemistry study of the bridged bis-alkylidene complex reveals their ligand-based reduction reactivity towards different oxidants such as hexachloroethane, disulfide and cyclooctatetraene.

## Abstract 2

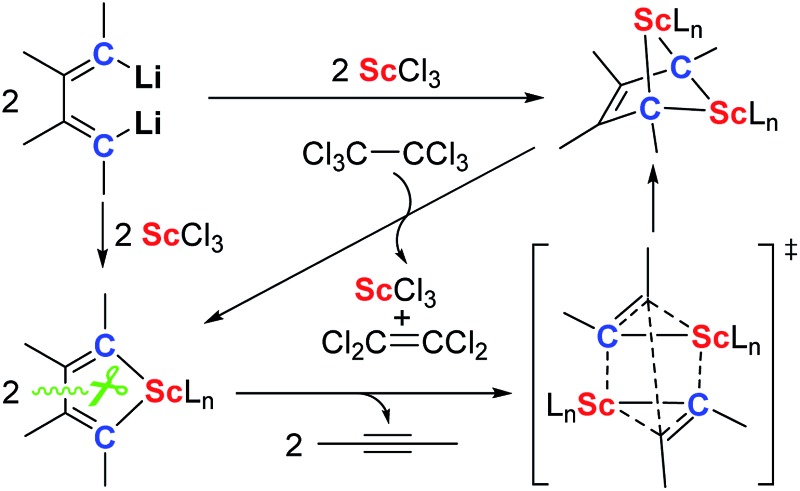
Bridged bis-alkylidene Sc(iii) complexes featuring a 2-butene-1,1,4,4-tetraanion are synthesized and show unexpected ligand-based two-electron or four-electron reduction reactivity towards different oxidants.

Transition metal carbene and alkylidene complexes have been extensively studied because of their importance in organometallic chemistry, coordination chemistry and synthetic organic chemistry.^1^ In contrast, rare-earth metal carbene and alkylidene complexes are very limited mainly due to the energy mismatch between the rare-earth metals and ligand orbitals.^2–12^ Since the rare-earth alkylidene complex was first postulated in 1979,^3^ pioneering works have been made to isolate and characterize it. Some pincer-like rare-earth alkylidene complexes have been reported independently by Cavell,^4^ Liddle,^5^ and Mézailles.^6^ Very recently, Cui *et al.* reported the lutetium methanediide-alkyl complexes,^7^ and Chen *et al.* reported the non-pincer-type mononuclear scandium alkylidene complexes.^8^ Furthermore, rare-earth methylidene complexes were also stabilized by chloride bridges^9^ or Lewis-acids such as AlMe_3_.^10^ Interestingly, mixed methyl/methylidene complexes^11^ and cubane-like methylidene complexes^12^ have been reported. Despite these recent advances, the chemistry of rare-earth alkylidene complexes is still in its infancy, and the bridged bis-alkylidene complex remains scarce.

Reductive reaction of rare-earth organometallic compounds is a fundamental process in organometallic chemistry and coordination chemistry.^13^ Rare earth metal complexes (Ce, Sm, Eu and Yb) supported by redox-inert ligands tend to perform a single electron redox process. The utilization of redox-active ligands at the rare earth metal centers is an alternative strategy for affording multi-electron redox reactivity.^14^ Ligand-based reductive chemistry of trivalent rare-earth organometallic compounds has received much attention. Evans and coworkers have made great progress in studying the reductive reactivity of (C_5_Me_5_)_3_Ln (Ln = La, Nd, Sm, *etc.*) and provided a wide variety of new reductive chemistry for rare earth metals.^13a,15^

Herein, we report the first synthesis of the bridged bis-alkylidene complex featuring a 2-butene-1,1,4,4-tetraanion and four Sc–C(sp^3^) bonds from 1,4-dilithio-1,3-butadienes and ScCl_3_. This reaction proceeds *via* two key intermediates: scandacyclopentadiene^16,17^ and scandacyclopropene.^18,19^ DFT calculations indicate that the dimerization of scandacyclopropenes *via* the cooperative double metathesis is the key factor for the formation of the bridged bis-alkylidene complex. Interestingly, the bridged bis-alkylidene scandium(iii) complex shows unexpected ligand-based two-electron or four-electron reduction reactivity towards different oxidants such as hexachloroethane, disulfide and cyclooctatetraene.

Silyl-substituted 1,4-dilithio-1,3-butadienes **1a–c** were readily prepared according to our previous procedure.^20^ When the 1 : 1 reaction of **1a** and solvated ScCl_3_ in THF was conducted at –20 °C, the light yellow crystalline complex **2a** could be isolated exclusively in 65% yield (Scheme 1). An X-ray analysis of **2a** revealed that it is a LiCl-ligated scandacyclopentadiene (Fig. 1). The Sc(iii) center adopts a distorted octahedral fashion bonded with two C(sp^2^) atoms, two chlorides and two THF molecules. The C1–C2 (1.348(4) Å) and C3–C4 (1.376(4) Å) bond lengths are within the range of standard C=C bond lengths, and the C2–C3 bond length (1.520(3) Å) indicates a typical C–C single bond. These data of bond lengths clearly show the butadienyl dianionic structure in **2a**.

**Scheme 1:**
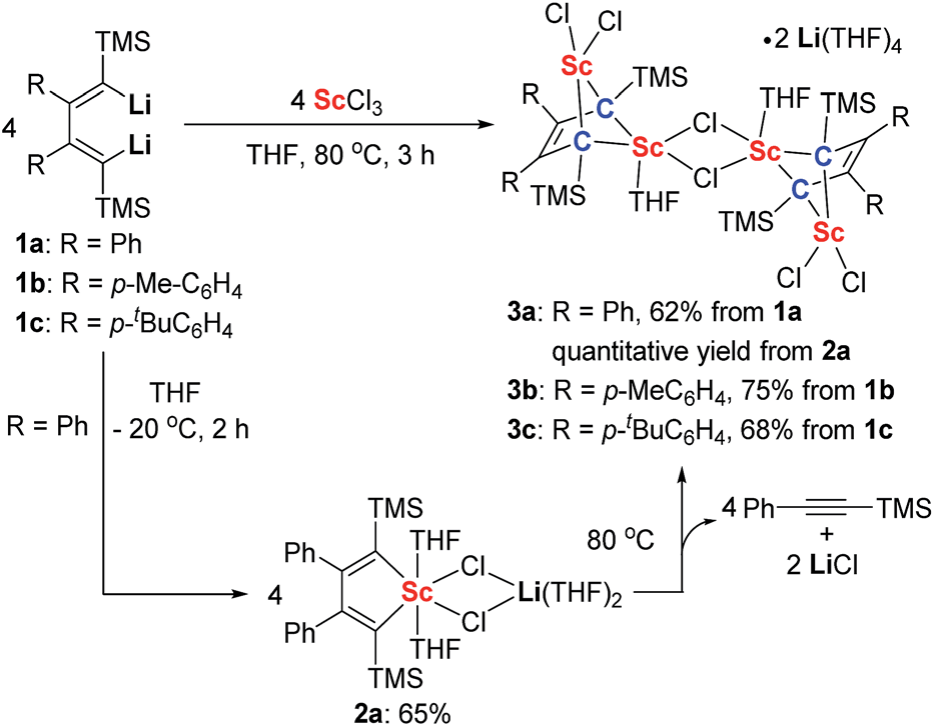
Synthesis of scandacyclopentadiene **2a** and bridged bis-alkylidene scandium(iii) complexes **3a–c**.

**Fig. 1:**
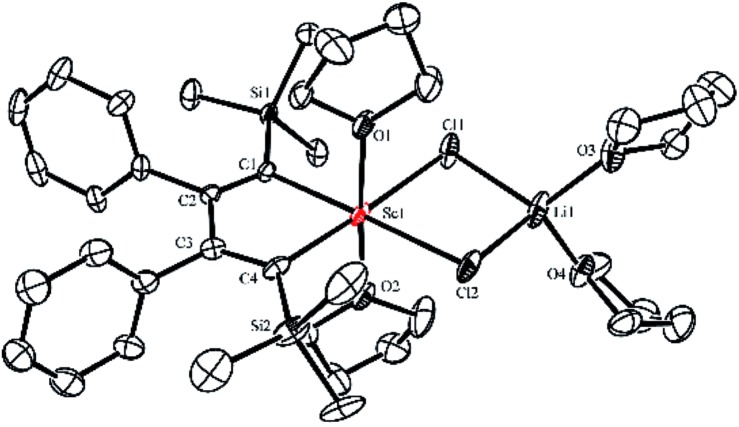
Molecular structure of complex **2a** with thermal ellipsoids at 30% probability. H atoms are omitted for clarity.

Complex **2a** is sensitive to air and moisture but stable under dry N_2_ atmosphere. In the ^1^H NMR spectrum in THF-*d*_8_, a singlet at –0.38 ppm was observed and assigned to the proton resonance of TMS groups. Two β-C(sp^2^) atoms (C2 and C3) displayed a singlet at 167.6 ppm in the ^13^C NMR spectrum, while two α-C(sp^2^) atoms (C1 and C4) showed a broad peak at 203.8 ppm, probably due to the coupling with scandium (nuclear spin quantum number *I* = 7/2). The ^1^H NMR spectrum of **2a** in THF-*d*_8_ showed no obvious change for 2 weeks at room temperature. However, when the THF-*d*_8_ solution of **2a** was heated at 45 °C for 3 h or 80 °C for 10 min, the TMS proton resonance at –0.38 ppm completely disappeared in the ^1^H NMR spectrum, and two new singlets integrated to the same number of protons appeared at –0.23 ppm and 0.20 ppm (see ESI† for more details). The singlet at 0.20 ppm was assigned to the TMS proton resonance of PhC≡CTMS by comparison with its standard spectrum. The GC retention time and molecular ion peak (*m*/*z* = 174) detected by GC-MS are also consistent with those of the standard sample of PhC≡CTMS. The other new singlet at –0.23 ppm was assigned to the TMS groups of a new complex **3a**, which was obtained in almost quantitative yield by thermolysis of **2a**. Furthermore, we found that the synthesis of **3a** does not require isolation of **2a** as the starting material. **3a** could be conveniently prepared by the reaction of **1a** with solvated ScCl_3_ in THF solution at 80 °C for 3 h. Similarly, **3b** and **3c** could be prepared from the corresponding 1,4-dilithio-1,3-butadienes and ScCl_3_ (Scheme 1).

An X-ray analysis of **3a** reveals it is a bridged bis-alkylidene complex and adopts a dimeric ate complex *via* μ_2_-chloride bridges (Fig. 2). One scandium center (*e.g.* Sc1) is bonded with two carbon atoms and two terminal chlorides, while the other one (*e.g.* Sc2) is bonded with two carbon atoms, two bridged chlorides and one THF. The Sc1–Sc2 distance (3.1366(9) Å) is the shortest length found in the literature, which is notably shorter than those in dinuclear scandium hydride complexes (3.20–3.40 Å).^21^ Two lithium atoms act as counterions, and each lithium atom forms a distorted tetrahedron surrounded by four THF molecules. The bond lengths of C1–C2 (1.468(4) Å) and C3–C4 (1.465(5) Å) in **3a** are significantly longer than those in **2a** [C1–C2, 1.348(4) Å; C(3)–C(4), 1.376(4) Å]. The bond length of C2–C3 (1.430(4) Å) in **3a** is significantly shorter than the corresponding C2–C3 (1.520(3) Å) in **2a**. Thus, the bond lengths in the C1–C2–C3–C4 moiety in **3a** are averaged and are not the classical bond lengths of C–C single and double bonds. These results show that **3a** has a highly delocalized structure with a tetraanionic ligand. Most importantly, these results are in striking contrast with what was observed previously for the transmetalation reactions of 1,4-dilithio-1,3-butadienes with metal salts which gave 1,3-butadiene-1,4-dianion complexes.^20^

**Fig. 2:**
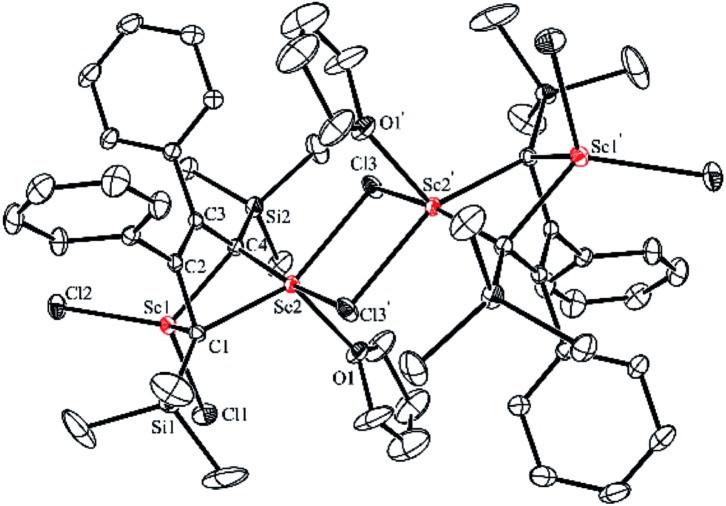
Molecular structure of complex **3a** with thermal ellipsoids at 30% probability. H atoms and two [Li(THF)_4_]^+^ counterions are omitted for clarity.

The formation of the asymmetric unit in **3a** from two molecules of **2a** along with elimination of two alkynes is a very interesting process and intrigued us to explore the reaction mechanism. The crossover reaction between **2a** and **2a-D_10_** was carried out. When the reaction mixture was quenched with H_2_O, **4a**, **4a-D_5_**, and **4a-D_10_** could all be detected by HRMS (Scheme 2). This result unambiguously reveals that the 2-butene-1,1,4,4-tetraanion moiety in **3a** should be originated from two molecules of scandacyclopentadienes instead of a simple reduction of a diene moiety in one scandacyclopentadiene. Thus, the crossover experiment excludes two possible pathways involving two eliminated alkynes from the same scandacyclopentadienes: (i) the cooperative intermolecular redox process, and (ii) stepwise intermolecular redox *via* the scandacyclopropene process (see ESI† for more details).

**Scheme 2:**
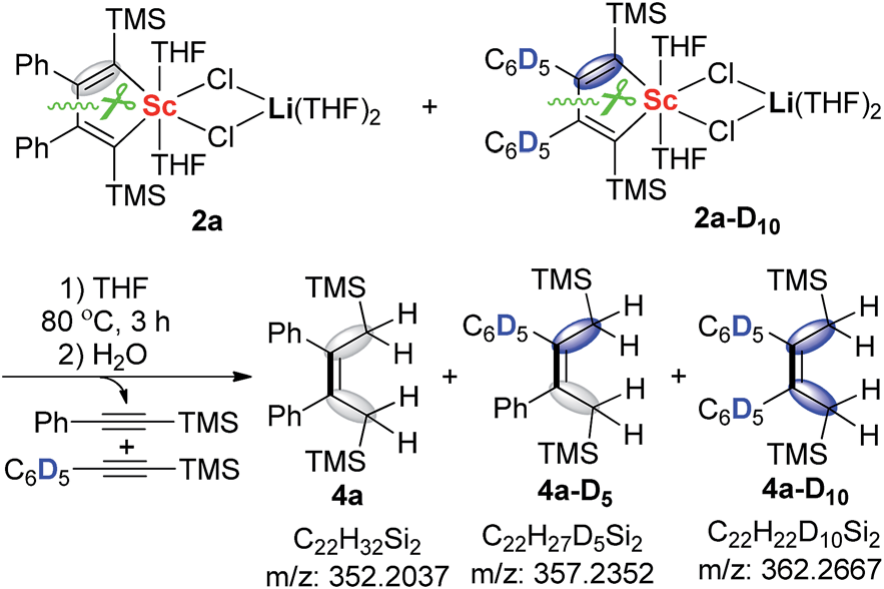
The crossover-reaction between **2a** and **2a-D_10_**.

Based on the above information, we proposed a mechanism involving the scandacyclopropene intermediate. For a better understanding of the formation of **3a**, DFT calculations were carried out using Gaussian 09 (Fig. 3).^22^ We chose the LiCl-free scandacyclopentadiene **IM1** as a starting model compound and the THF-ligated monomer **3a-M** as a targeted compound for simplicity.^23^ The structures of all of the minima and transition states were optimized at the B3LYP^24^/LANL2DZ (for Sc)/6-31+G* (for other elements) level in the gas phase. The effect of the solvent was examined by performing single-point self-consistent reaction field (SCRF) calculations based on the polarizable continuum model (PCM) for gas-phase optimized structures. Scandacyclopentadiene **IM1** will undergo β,β′-C–C bond cleavage to generate scandacyclopropene **IM2** by release of one equiv. of alkyne. The β,β′-C–C bond cleavage from **IM1** to **IM2** is the critical step with the highest energy barrier of 13.3 kcal mol^–1^ in the solution phase, which means that **IM1** is isolable. Metallacyclopropenes, as an important class of reactive intermediate, have been isolated and characterized in transition and main group organometallic chemistry.^18,19^ The metallacyclopropene, *e.g.* aluminacyclopropene, can undergo dimerization to give a 1,4-dialuminacyclohexadiene.^25^ In contrast, rare-earth metallacyclopropenes are unknown. **IM2** is the first optimized structure of a rare-earth metallacyclopropene by DFT calculations. Next, we tried to optimize the dimeric structure of **IM3′** which is similar to 1,4-dialuminacyclohexadiene. However, the optimization of the structure of **IM3′** to a local energy minimum failed, probably because of its high energy and instability. Rather than giving **IM3′**, a new intermediate, **IM3**, resulting from two **IM2** species approaching each other *via* the weak Sc–C interaction, was optimized to a local minimal energy, 2.7 kcal mol^–1^ lower than **IM2**. Surprisingly, a cooperative double metathesis of **IM3** gives **3a-M** *via* the transition state **TS2**. In **TS2**, two scandacyclopropene rings adopt a triangular prism geometry, in which each Sc atom is coordinated to another carbon neighbouring TMS group. This geometry of **TS2** could also explain the selectivity of C(Ph)–C(Ph) coupling.

**Fig. 3:**
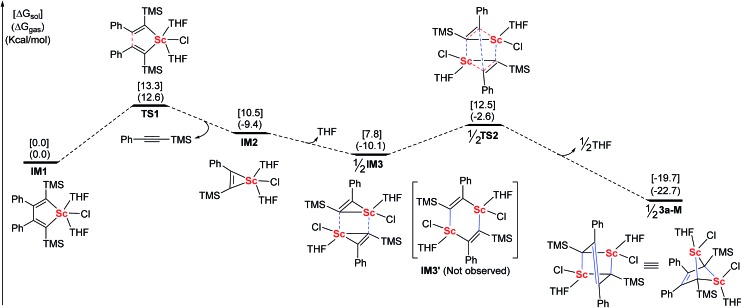
DFT calculated energy profiles of related intermediates and transition-states in the generation of **3a-M** (red lines: broken bonds; blue lines: newly formed bonds).

The structure of **3a** features the 2-butene-1,1,4,4-tetraanion moiety and thus we thought it could be oxidized to generate the diene moiety in **2a**, as illustrated in Scheme 3. As we expected, **2a** was generated by treatment with two equivalents of hexachloroethane as an oxidant (Scheme 3a). This reaction resulted in the formation of ScCl_3_ which can be characterized as a ScCl_3_(THF)_3_ adduct by X-ray analysis, along with two equivalents of tetrachloroethylene which were identified using the ^13^C NMR spectrum and GC-MS. When four equivalents of hexachloroethane were used and the reaction mixture was heated at 80 °C, **3a** was transformed to PhC≡CTMS and ScCl_3_ (Scheme 3b). Furthermore, when disulfide **5** served as an oxidant,^26^ the reaction of **3a** with **5** provided complex **6** (see ESI† for the X-ray structure of **6**, Scheme 3c) along with the formation of PhC≡CTMS. When **3a** was treated with cyclooctatetraene at 80 °C, cyclooctatetraene was reduced to the cyclooctatetraene dianion. The corresponding complex **7** (see ESI† for the X-ray structure of **7**, Scheme 3d) could be isolated after being recrystallized in DME (DME = 1,2-dimethoxyethane) in high yields along with the formation of PhC≡CTMS. These results clearly show that the bridged bis-alkylidene scandium(iii) complex **3a** can act as an efficient two-electron or four-electron reductant.

**Scheme 3:**
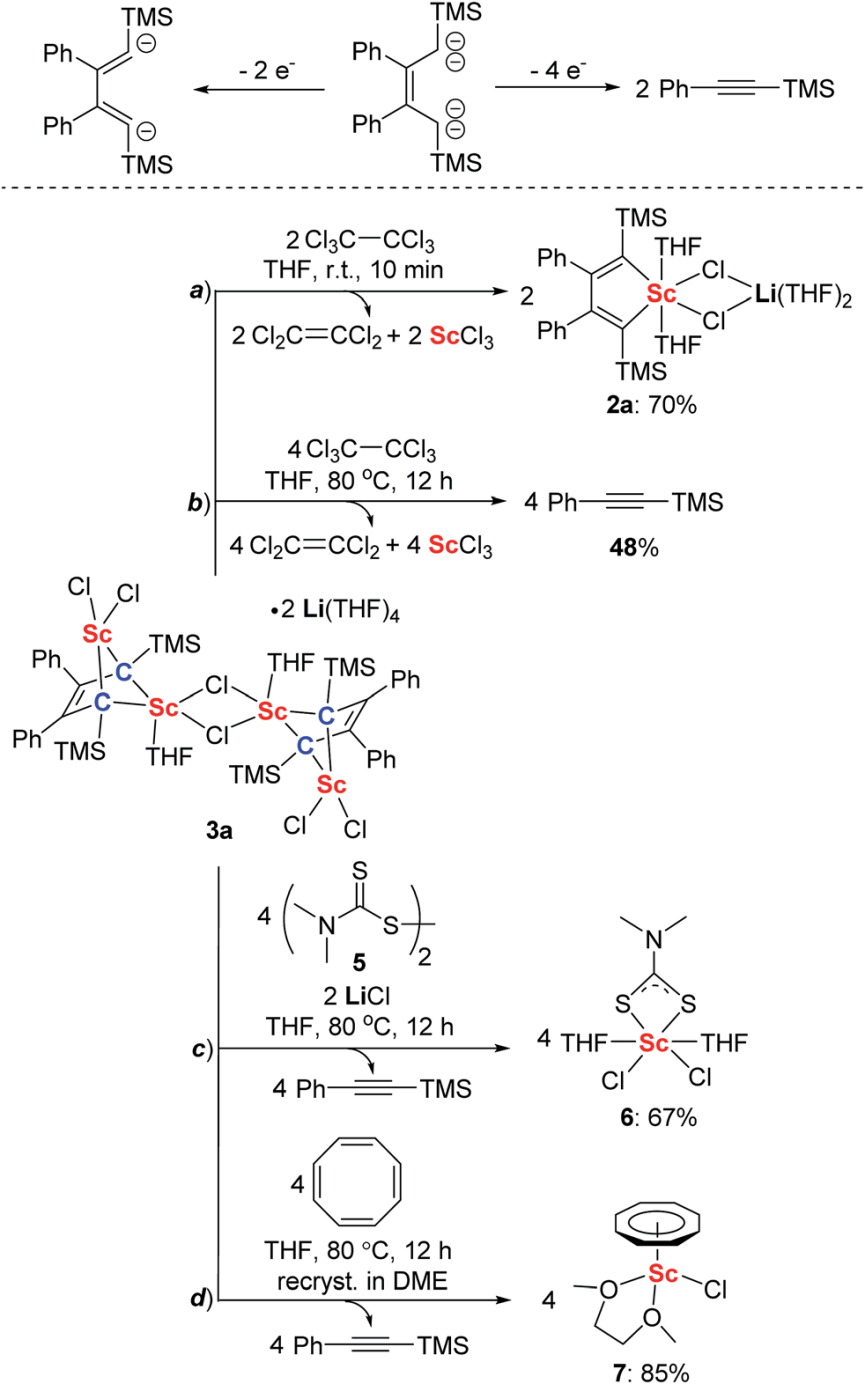
Ligand-based reduction reactivity of **3a** towards different oxidants.

## Conclusions

In summary, we have developed a simple and efficient synthetic method for the first series of well-defined bridged bis-alkylidene scandium(iii) complexes from 1,4-dilithio-1,3-butadienes and ScCl_3_. This reaction proceeds *via* two key intermediates: an isolable scandacyclopentadiene and a proposed scandacyclopropene. A mechanistic pathway of C–C bond recombination through the dimerization of scandacyclopropene intermediates is elucidated well by DFT calculations. Bridged bis-alkylidene scandium(iii) complexes are found to show ligand-based reduction reactivity towards different kinds of oxidant. Further reaction chemistry of bis-alkylidene scandium(iii) complexes and characterization of scandacyclopropenes are in progress.

## Conflicts of interest

There are no conflicts of interest to declare.

## References

[cit1] Schrock R. R. (2006). Angew. Chem., Int. Ed..

[cit2] Arnold P. L., Casely I. J. (2009). Chem. Rev..

[cit3] Schumann H., Müller J. (1979). J. Organomet. Chem..

[cit4] Aparna K., Ferguson M., Cavell R. G. (2000). J. Am. Chem. Soc..

[cit5] Mills D. P., Soutar L., Lewis W., Blake A. J., Liddle S. T. (2010). J. Am. Chem. Soc..

[cit6] Fustier M., Le Goff X. F., Le Floch P., Mézailles N. (2010). J. Am. Chem. Soc..

[cit7] Li S., Wang M., Liu B., Li L., Cheng J., Wu C., Liu D., Liu J., Cui D. (2014). Chem.–Eur. J..

[cit8] Mao W., Xiang L., Lamsfus C. A., Maron L., Leng X., Chen Y. (2017). J. Am. Chem. Soc..

[cit9] Dietrich H. M., Törnroos K. W., Anwander R. (2006). J. Am. Chem. Soc..

[cit10] Huang W., Carver C. T., Diaconescu P. L. (2011). Inorg. Chem..

[cit11] Hong J., Zhang L., Yu X., Li M., Zhang Z., Zheng P., Nishiura M., Hou Z., Zhou X. (2011). Chem.–Eur. J..

[cit12] Zhang W.-X., Wang Z., Nishiura M., Xi Z., Hou Z. (2011). J. Am. Chem. Soc..

[cit13] (b) Crabtree R. H., The Organometallic Chemistry of the Transition Metals, Wiley Interscience, New York, 2014.

[cit14] Lv Y., Kefalidis C. E., Zhou J., Maron L., Leng X., Chen Y. (2013). J. Am. Chem. Soc..

[cit15] Evans W. J., Davis B. L. (2002). Chem. Rev..

[cit16] (c) Takahashi T. and Li Y., in Titanium and Zirconium in Organic Synthesis, ed. I. Marek, Wiley-VCH, Weinheim, 2002, ch. 2.

[cit17] Xu L., Wang Y., Wang Y.-C., Wang Z., Zhang W.-X., Xi Z. (2016). Organometallics.

[cit18] Parker K. D. J., Fryzuk M. D. (2015). Organometallics.

[cit19] Zhang L., Hou G., Zi G., Ding W., Walter M. D. (2016). J. Am. Chem. Soc..

[cit20] Xi Z. (2010). Acc. Chem. Res..

[cit21] Halcovitch N. R., Fryzuk M. D. (2013). Organometallics.

[cit22] M. J. Frisch, *et al.*, Gaussian 09 (Revision C.01), Gaussian, Inc., Wallingford CT, 2010, for full reference, see ESI.

[cit23] The Sc–O interaction is much stronger than the Sc–Cl interaction because of the oxophilicity of rare-earth elements. In THF, LiCl in **2a** will be easily replaced by THF to yield a LiCl-free complex **IM1**. Based on the effects of the THF atmosphere, we excluded LiCl from the calculation. In THF, **3a** tends to be a monomer due to the solvent coordination interaction. Furthermore, the bond lengths and angles of the calculated monomeric structure are similar to those of the crystal dimeric structure. Thus, we think the calculation of monomer **3a-M** is enough to describe the reaction pathway

[cit24] Becke A. D. (1993). J. Chem. Phys..

[cit25] Üffing C., Ecker A., Köppe R., Merzweiler K., Schnöckel H. (1998). Chem.–Eur. J..

[cit26] Basalov I. V., Lyubov D. M., Fukin G. K., Shavyrin A. S., Trifonov A. A. (2012). Angew. Chem., Int. Ed..

