# Supplementary material for: Formation and ligand-based reductive chemistry of bridged bis-alkylidene scandium(iii) complexes
Source: Chem Sci. 2017 Jul 13;8(10):6852–6. doi: 10.1039/c7sc02018j (PMC5632790; doi:10.1039/c7sc02018j)
Supplement: Supplementary file 1 [file SC-008-C7SC02018J-s001.pdf]

Electronic Supplementary Material (ESI) for Chemical Science.
This journal is © The Royal Society of Chemistry 2017

Cover Page for Supporting Information

Manuscript Title:

Formation and Ligand-Based Reductivity of Bridged Bis-alkylidene Scandium(III) Complexes

Authors:

Wangyang Ma,[a] Chao Yu,[a] Yue Chi,[a] Tianyang Chen,[a] Lianjun Wang,[ac] Jianhao Yin,[a] Baosheng Wei,[a] Ling Xu,[a] Wen-Xiong Zhang,*[ab] and Zhenfeng Xi[a]

Affiliations:

[a] Beijing National Laboratory for Molecular Sciences (BNLMS), Key Laboratory of Bioorganic Chemistry and Molecular Engineering of Ministry of Education, College of Chemistry, Peking University, Beijing 100871, China

[b] State Key Laboratory of Elemento-Organic Chemistry, Nankai University, Tianjin 300071, China

[c] School of Chemistry and Chemical Engineering, Hunan Institute of Engineering, Xiangtan, 411104, China

Contents:

1) Experimental Details and Characterization Data S2

2) Copies of ^{1}H NMR and ^{13}C NMR Spectra of All New Compounds S7

3) X-ray Crystallographic Studies S17

4) Mechanistic Investigations for the Formation of 3a S23

***In situ* ^{1}H NMR Monitoring the Transformation from 2a to 3a and Alkyne S23**

***In situ* ^{1}H NMR Monitoring the Transformation from 3a to 2a S23**

***In situ* ^{1}H NMR Monitoring the Transformation from 3a to Alkyne S24**

The Crossover Reaction among 2a and 2a-D_{10} S25

Proposed Mechanisms for the Formation of 3a from 2a S26

5) Details of DFT Calculations S28

6) References S43

1) Experimental Details and Characterization Data

Unless otherwise noted, all reactions were conducted under slightly positive dry nitrogen pressure using standard Schlenk line techniques or under a nitrogen atmosphere in a Vigor (SG 1200/750TS-F) glovebox. The nitrogen in the glove box was constantly circulated through a copper/molecular sieves catalyst unit. The oxygen and moisture concentrations in the glovebox atmosphere were monitored by an O_2/H_2O Combi-Analyzer to ensure both were always below 1 ppm. Unless otherwise noted, all starting materials were commercially available and were used without further purification. Solvents were purified by an Mbraun SPS-800 Solvent Purification System and dried over fresh Na chips in a glovebox. Organometallic samples for NMR spectroscopic measurements were prepared in a glovebox by the use of J.Young valve NMR tubes (Wilmad 528-JY). ^{1}H, and ^{13}C NMR spectra were recorded on 400 MHz spectrometer (FT, 400 MHz for ^{1}H; 100 MHz for ^{13}C) at room temperature, unless otherwise noted. Mass spectra (MS) were recorded on a GC/MS spectrometer using an EI source. High-resolution mass spectra (HRMS) were recorded on FTMS mass spectrometer using an EI source or ESI source. Elemental analyses were performed on a Vario MICRO cube elemental analyzer.

Note: The signals of solvents in NMR spectra are due to the vapor of solvents in the glovebox. Because of the high sensitivity of these organometallics to air and moisture, some samples easily lost weight even in double layers of tin boats. Thus their elemental analysis data are not available.

1,4-Diiodo-1,3-butadienes **1a'-1c'** and **1a-D$_{10}$'** were synthesized by the reported procedure.[1,2] **1c'** and **1a-D$_{10}$'** are new compounds.

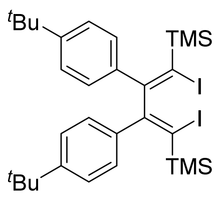

1c'

1c': Light yellow solid, isolated yield 64% (12.0 mmol scale, 5.5 g). ^{1}H NMR (400 MHz, C_6D_6) δ 0.11 (s, 18H, Me_3Si), 1.10 (s, 18H, ^{t}Bu,), 7.05 (d, J = 8.6 Hz, 4H, C_6H_5), 7.36 (d, J = 7.9 Hz, 4H, C_6H_5). ^{13}C NMR (100 MHz, C_6D_6) δ 1.1, 31.3, 34.6, 112.6, 125.1, 129.6, 137.1, 151.7, 164.0. HRMS (ESI, m/z) calcd for $C_{30}H_{45}I_2Si_2$ [M + H]$^+$: 715.1144, found 715.1140.

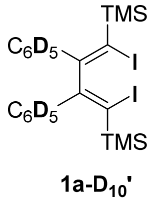

1a-D$_{10}$'

1a-D$_{10}$': Light green solid, isolated yield 45% (30.0 mmol scale, 8.3 g). ^{1}H NMR (400 MHz, THF-d^8) δ -0.04 (s, 18H, Me_3Si). ^{13}C NMR (100 MHz, THF-d^8) δ 1.0, 112.7, 128.1 (t, $J_{C\text{-}D}$ = 24.1 Hz), 128.6 (t, $J_{C\text{-}D}$ = 24.2 Hz), 129.7 (t, $J_{C\text{-}D}$ = 24.2 Hz), 140.2, 164.3.

General Procedure for the Preparation of 1,4-Dilithio-1,3-butadienes 1a-c and 1a-D_{10}

^{t}BuLi (4.0 mmol, 1.3 M in pentane) was added dropwise at -78 °C to a stirred solution of 1,4-diiodo-1,3-butadienes **1a'-c'** and **1a-D_{10}'** (1.0 mmol) in Et_2O (10 mL). After stirred at -78 °C for 1 h, the reaction mixture was allowed to warm to room temperature. The solvents were removed under vacuum in the glovebox and 1,4-dilithio-1,3-butadienes **1a-c** were used for next step without further purification. The pure **1a-D_{10}** was obtained by extracting the *in situ*-generated **1a-D_{10}** with hexane and recrystallization in hexane after filtration of LiI.

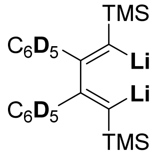

1a-D_{10}

1a-D_{10}: Red powder, isolated yield 98% (365.1 mg). ^{1}H NMR (400 MHz, THF-d^8) δ -0.34 (s, 18H, Me_3Si). ^{13}C NMR (100 MHz, THF-d^8) δ 3.1, 122.4 (t, J_{C-D} = 23.0 Hz), 126.0 (t, J_{C-D} = 23.1 Hz), 129.0 (t, J_{C-D} = 23.3 Hz), 154.0, 164.1, 208.1.

General Procedure for the Synthesis of Scandacyclopentadiene 2a and 2a-D_{10}

In the glovebox, $ScCl_3$ (151.3 mg, 1.0 mmol) were dissolved in cold (-20 °C) THF solution (5 ml) in a 25 mL flask. And then the cold THF solution (10 mL) of 1,4-dilithio-1,3-butadiene **1a** (362.5 mg, 1.0 mmol) was added dropwise into the above solution and stirred at -20 °C for 2 h. After the stirring is finished, the solvents were removed under reduced pressure. The residue was extracted with cold toluene for several times, LiCl was filtered and the volatiles of the filtrate were removed under reduced pressure to give dark yellow solids. Complex **2a** could be isolated as yellow crystalline powder in good yield upon recrystallization of the saturated THF/Et_2O solution. The single crystals of **2a** suitable for X-ray analysis could be obtained by volatilization of THF/Et_2O solution at -20 °C for 2 days.

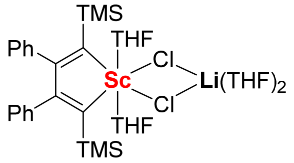

2a

2a: Yellow solid, isolated yield 65% (493.9 mg). ^{1}H NMR (400 MHz, THF-d^8) δ -0.38 (s, 18H, Me_3Si), 6.55-6.57 (m, 4H, C_6H_5), 6.60-6.65 (m, 2H, C_6H_5), 6.69-6.72 (m, 4H, C_6H_5). ^{13}C NMR (100 MHz, THF-d^8) δ 2.6, 26.4, 68.4, 123.9, 126.8, 129.9, 150.5 167.6, 203.8.

In the glovebox, $ScCl_3$ (151.3 mg, 1.0 mmol) were dissolved in cold (-20 °C) THF solution (5 ml) in a 25 mL flask. And then the cold THF solution (10 mL) of deuterated 1,4-dilithio-1,3-butadiene **1a-D_{10}** (372.5 mg, 1.0 mmol) was added dropwise into the above solution and stirred at -20 °C for 2 h. After the

stirring is finished, the solvents were removed under reduced pressure. The residue was extracted with cold toluene for several times, LiCl was filtered and the volatiles of the filtrate were removed under reduced pressure to give dark yellow solids. Complex **2a-D$_{10}$** could be isolated as yellow crystalline powder in good yield upon recrystallization of the saturated THF/Et_2O solution.

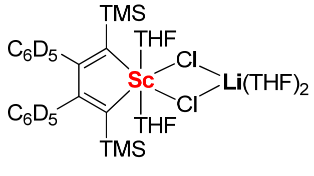

2a-D$_{10}$

2a-D$_{10}$: Yellow solid, isolated yield 62% (477.3 mg). ^{1}H NMR (500 MHz, THF-d^8) δ -0.37 (s, 18H, Me_3Si). ^{13}C NMR (125 MHz, THF-d^8) δ 2.6, 123.4 (t, $J_{C\text{-}D}$ = 24.2 Hz), 126.3 (t, $J_{C\text{-}D}$ = 24.4 Hz), 129.4 (t, $J_{C\text{-}D}$ = 24.2 Hz), 150.3 167.6, 203.8.

General Procedure for the Syntheses of Organo-di-scandium complexes 3a-c

In the glovebox, 1,4-dilithio-1,3-butadiene **1** (1.0 mmol) was dissolved in THF (10 mL) in a 25 mL sealed tube. Then THF suspension of $ScCl_3$ (151.3 mg, 1.0 mmol) was added at room temperature. And then the sealed tube was transferred outside of the glovebox and heated over 80 °C for 3 h in an oil bath (it is noteworthy that **2** could transform quantitatively into **3** at 45 °C for 3 h or 80 °C for 10 min in 0.05 mmol scale based on our i*n situ* ^{1}H NMR monitoring illustrated below, however, longer reaction time are required for complete transformation in 1.0 mmol scale). After the heat is finished, the sealed tube was transferred back to the glovebox and poured into a 25 ml flask. The solvents were removed under reduced pressure. The residue was extracted with toluene for several times, LiCl was filtered and the volatiles of the filtrate were removed under reduced pressure to give crimson solids. Complexes **3a-c** could be isolated as red crystalline powder in general to good yields upon recrystallization of the saturated THF/Et_2O solution. The single crystals of **3a** suitable for X-ray analysis could be obtained by volatilization of THF/Et_2O solution at room temperature for 1 day.

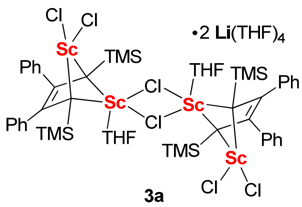

3a

3a: Red solid, isolated yield 62% (282.8 mg). ^{1}H NMR (400 MHz, THF-d^8) δ -0.23 (s, 36H, Me_3Si), 6.67 (t, J = 7.3 Hz, 4H, C_6H_5), 6.78 (t, J = 7.5 Hz, 8H, C_6H_5), 7.52 (d, J = 7.3 Hz, 8H, C_6H_5). ^{13}C NMR (100 MHz, THF-d^8) δ 5.2, 26.4, 68.3, 123.9, 126.5, 131.8, 134.4, 148.9.

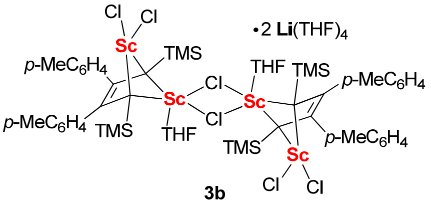

3b

3b: Red solid, isolated yield 75% (352.5 mg). ^{1}H NMR (400 MHz, THF-d^{8}) δ -0.16 (s, 36H, Me_3Si), 2.09 (s, 12H, CH_3), 6.65 (d, J = 7.9 Hz, 8H, C_6H_5), 7.53 (d, J = 7.9 Hz, 8H, C_6H_5). ^{13}C NMR (100 MHz, THF-d^{8}) δ 5.6, 21.3, 26.4, 68.3, 127.2, 132.1, 132.9, 135.4, 144.8.

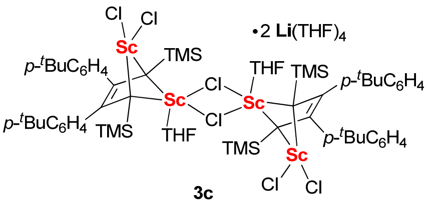

3c

3c: Red solid, isolated yield 68% (348.4 mg). ^{1}H NMR (400 MHz, THF-d^{8}) δ -0.18 (s, 36H, Me_3Si), 1.12 (s, 36H, ^{t}Bu), 6.82 (d, J = 8.0 Hz, 8H, C_6H_5), 7.51 (d, J = 8.0 Hz, 8H, C_6H_5). ^{13}C NMR (100 MHz, THF-d^{8}) δ 5.5, 26.4, 31.9, 34.6, 68.4, 122.9, 131.8, 135.5, 144.9, 146.2.

General Procedure for the Synthesis of 6

In the glovebox, a THF solution (~10 mL) of tetramethylthiuram disulfide (480.8 mg, 2.0 mmol) and LiCl (42.4 mg, 1.0 mmol) was added to a solution of **3a** (912.4 mg, 0.5 mmol) in THF (~10 mL) in a 25 mL sealed tube. And then the sealed tube was transferred outside of the glovebox and heated over 80 °C for 12 h in an oil bath. After the heat is finished, the sealed tube was transferred back to the glovebox and poured into a 25 ml flask. The solvent THF was removed under reduced pressure and hexane (~5 mL) was added to the residue. The mixture was stored at -20 °C overnight. Then the yellow solution was removed and the colourless powder was washed with cold hexane (~5 mL). The residual powder was dried under a vacuum. The single crystals suitable for X-ray analysis could be grown from THF/Hexane solution of **6** for 1 week.

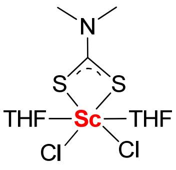

6

6: Colourless solid, isolated yield 67% (509.6 mg). ^{1}H NMR (400 MHz, THF-d^{8}) δ 3.40 (s, 6H, NMe_2). ^{13}C NMR (100 MHz, THF-d^{8}) δ 26.4, 41.8, 68.2. It is noteworthy that **6** is sparingly soluble in THF-d_8, so the ^{13}C NMR signals of amidinato carbon is not observable even after 12 h of measuring time. Anal. Calcd for $C_{11}H_{22}Cl_2NO_2S_2Sc$: C, 34.74; H, 5.83, N, 3.68. Found: C, 34.44; H, 5.87, N, 3.69.

General Procedure for the Synthesis of 7

In the glovebox, a THF solution (~10 mL) of cyclooctatetraene (104.2 mg, 1.0 mmol) was added to a solution of **3a** (456.2 mg, 0.25 mmol) in THF (~10 mL) in a 25 mL sealed tube. And then the sealed tube was transferred outside of the glovebox and heated over 80 °C for 12 h in an oil bath. After the heat is finished, the sealed tube was transferred back to the glovebox and poured into a 25 ml flask. The solvent THF was removed under reduced pressure and hexane (~5 mL) was added to the residue. The mixture was stored at -20 °C overnight. Then the yellow solution was removed and the pale yellow powder was washed with cold hexane (~5 mL). The residual powder was dried under a vacuum. The single crystals suitable for X-ray analysis could be grown from DME (DME = 1,2-dimethoxyethane)/THF/Hexane solution of **7** for 3 days.

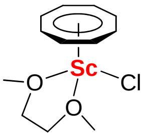

7

7: Pale yellow solid, isolated yield 85% (233.5 mg). ^{1}H NMR (400 MHz, THF-d^8) δ 3.27 (s, 6H, OMe), 3.43 (s, 4H, CH_2), 6.53 (s, 8H, CH) ^{13}C NMR (100 MHz, THF-d^8) δ 58.9, 72.7, 96.5.

2) Copies of ^{1}H NMR and ^{13}C NMR Spectra of All New Compounds

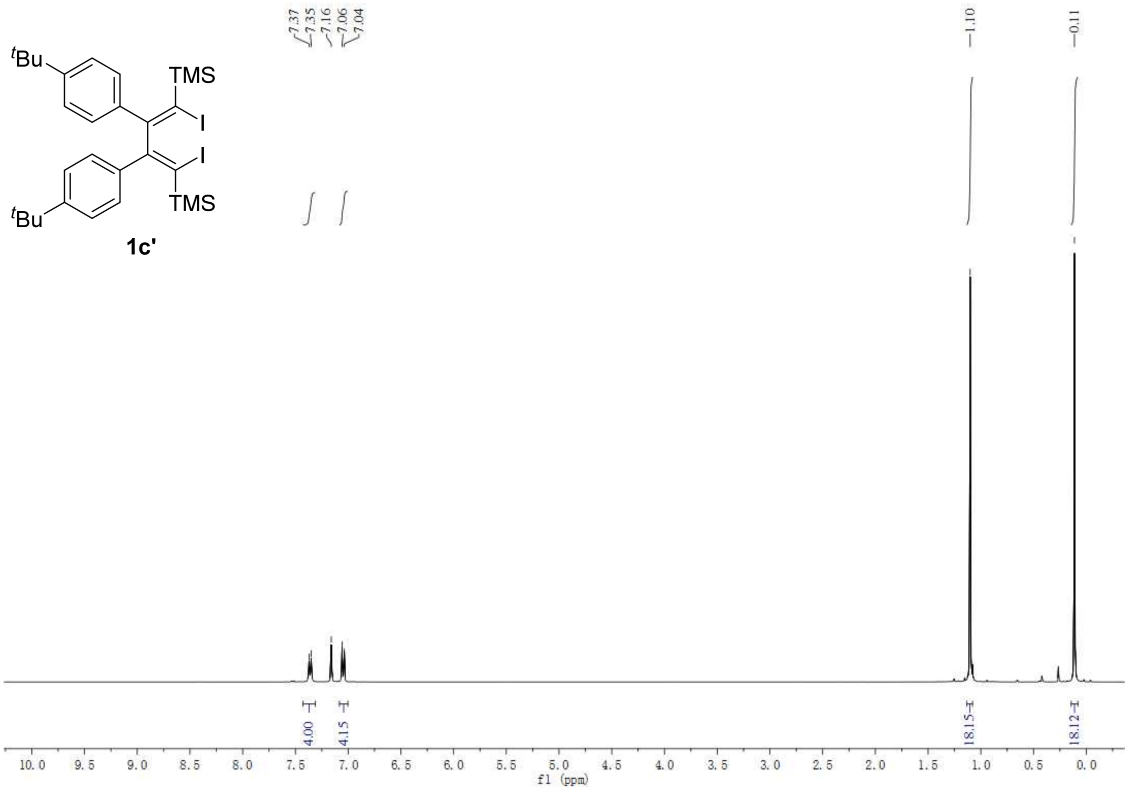

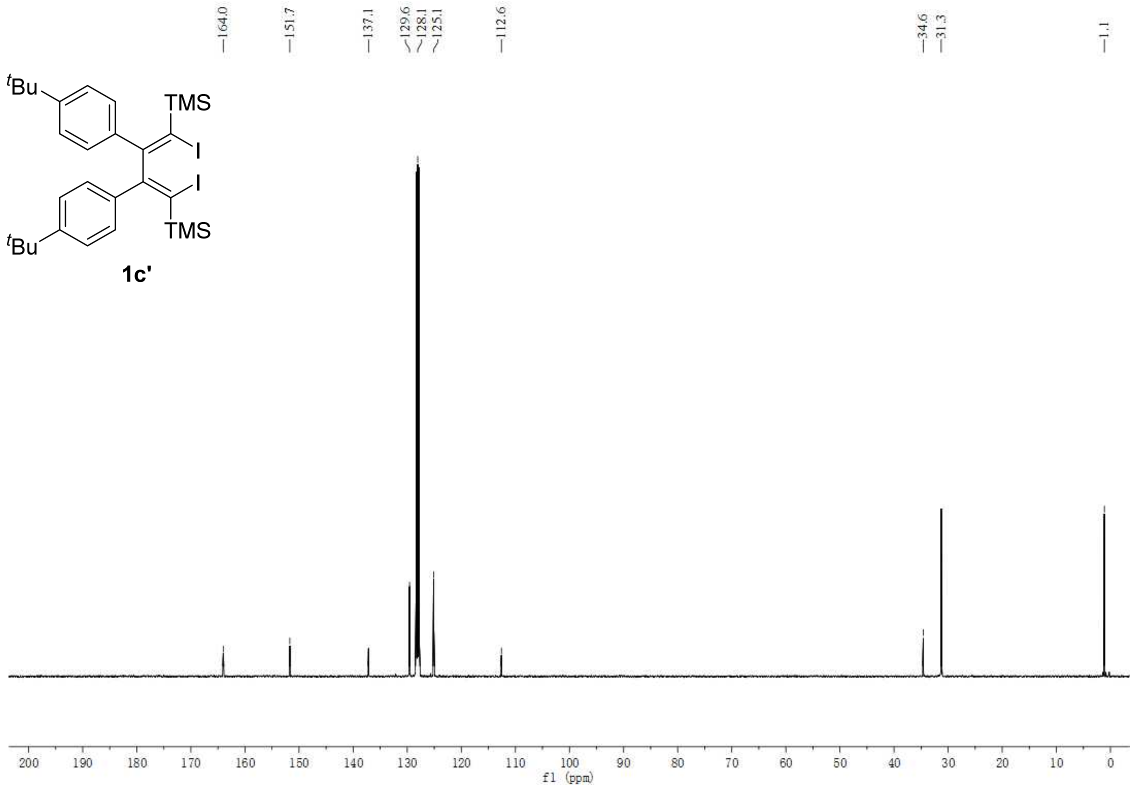

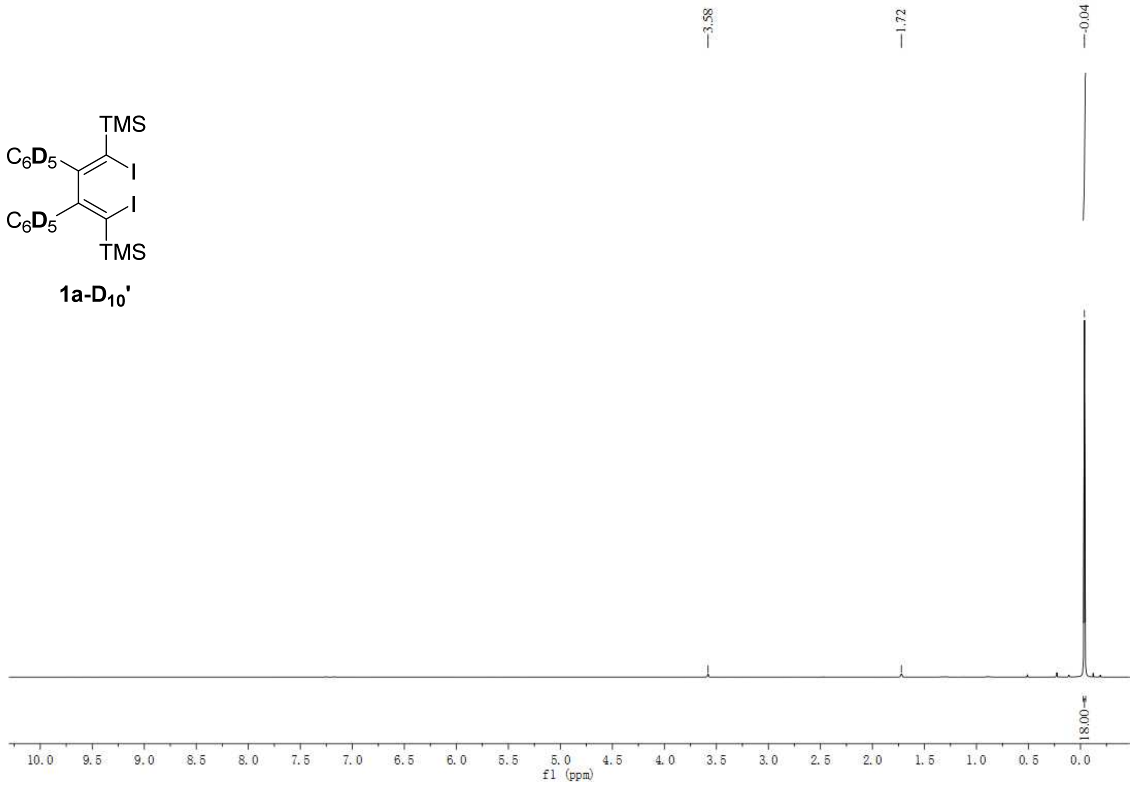
TMS
C_6D_5
I
I
C_6D_5
TMS
1a-D_{10}'
3.58
1.72
-0.04
18.00
f1 (ppm)

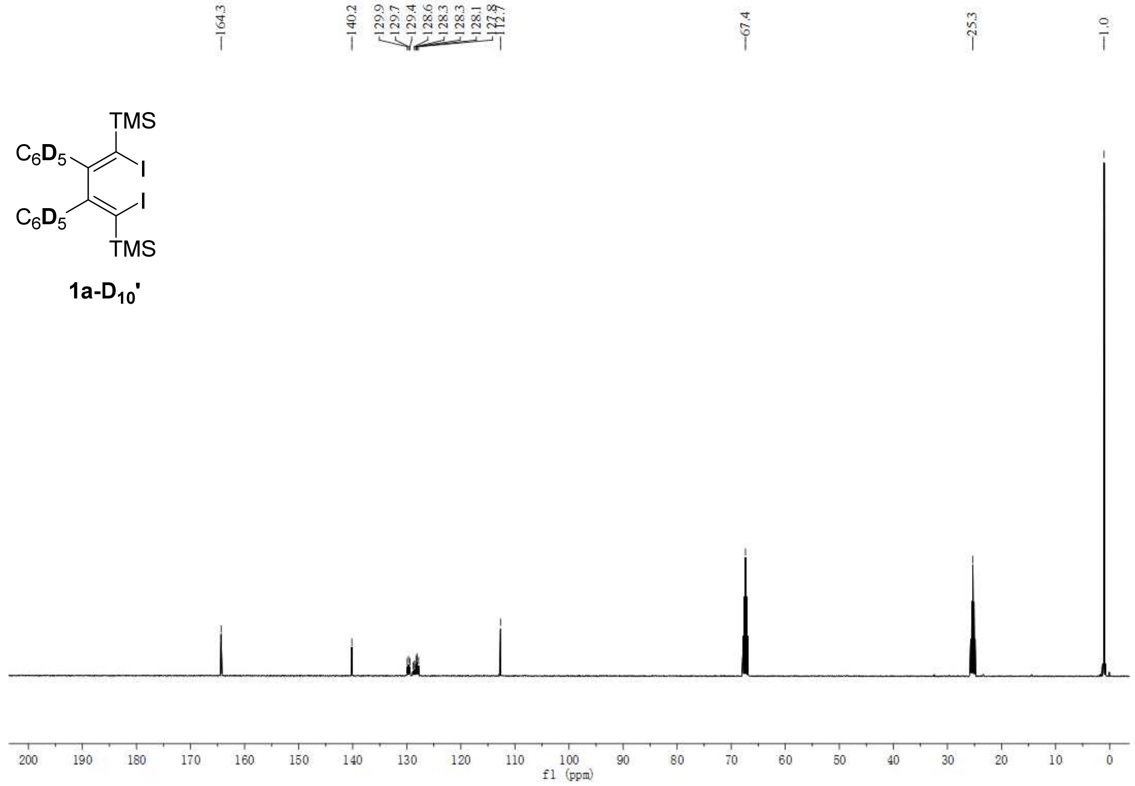
TMS
C_6D_5
I
I
C_6D_5
TMS
1a-D_{10}'
164.3
140.2
129.9
129.7
129.4
128.6
128.3
128.3
128.1
127.8
112.7
67.4
25.3
1.0
f1 (ppm)

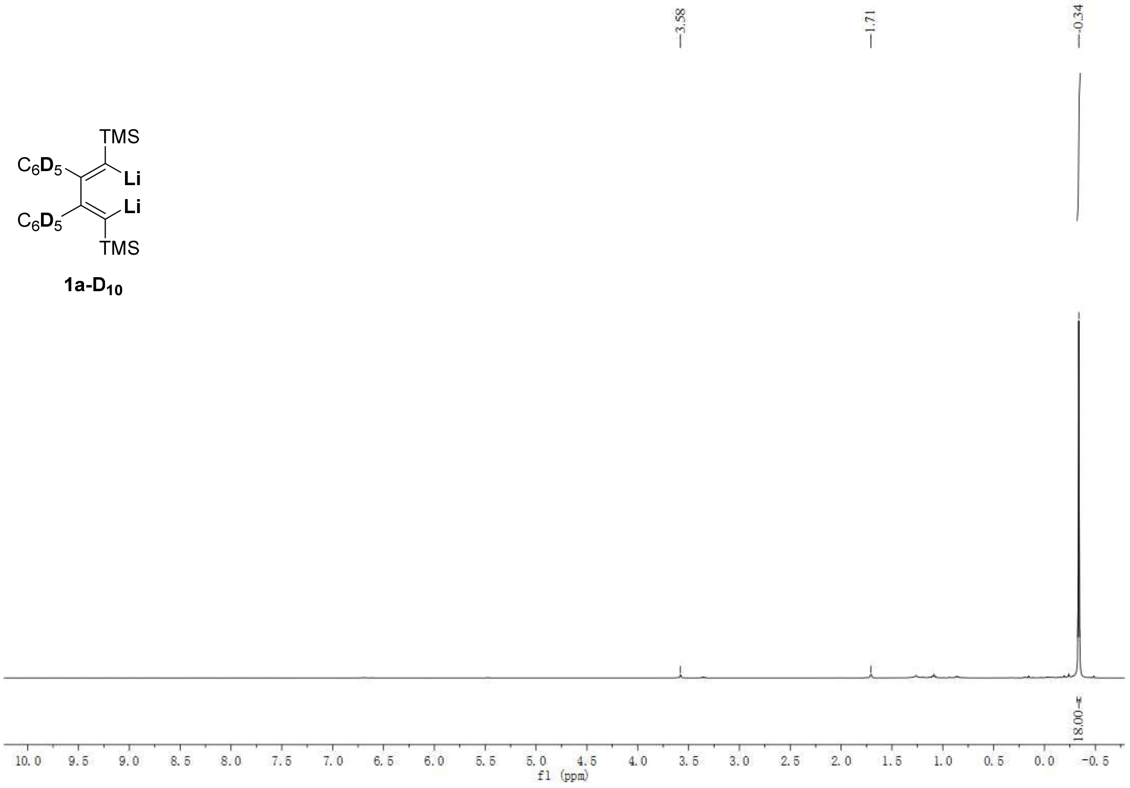

3.58
1.71
0.34
TMS
C6D5
Li
Li
C6D5
TMS
1a-D10
18.00
10.0 9.5 9.0 8.5 8.0 7.5 7.0 6.5 6.0 5.5 5.0 4.5 4.0 3.5 3.0 2.5 2.0 1.5 1.0 0.5 0.0 -0.5
f1 (ppm)

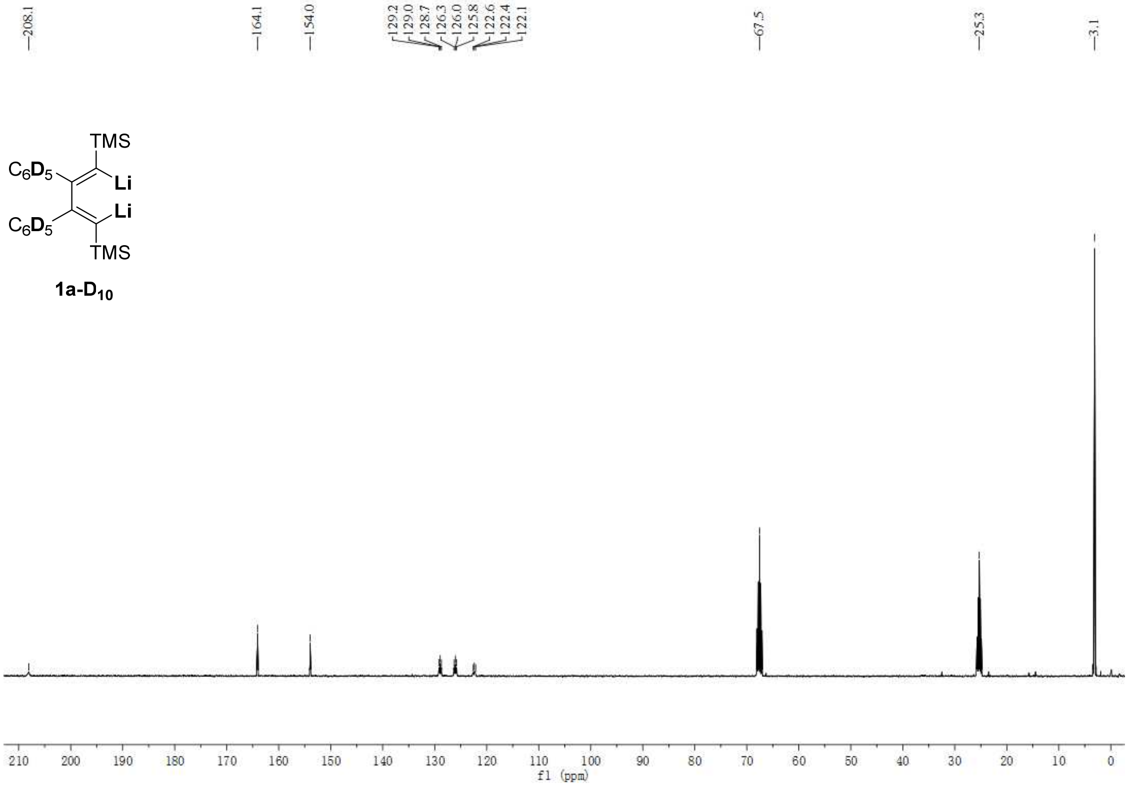

208.1
164.1
154.0
129.2
129.0
128.7
126.3
126.0
125.8
122.6
122.4
122.1
67.5
25.3
3.1
TMS
C6D5
Li
Li
C6D5
TMS
1a-D10
210 200 190 180 170 160 150 140 130 120 110 100 90 80 70 60 50 40 30 20 10 0
f1 (ppm)

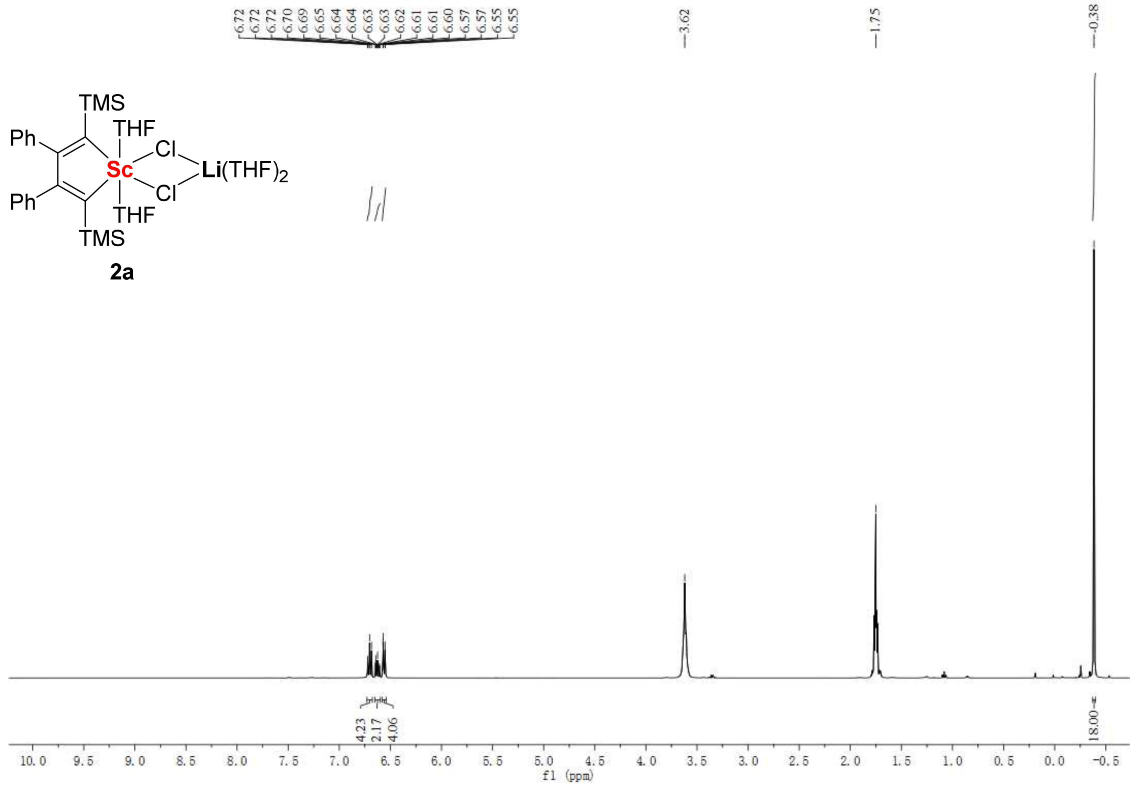
TMS
THF
Ph
Cl
Sc
Li(THF)2
Ph
Cl
THF
TMS
2a
6.72 6.72 6.72 6.70 6.69 6.65 6.64 6.64 6.63 6.63 6.62 6.61 6.61 6.60 6.57 6.57 6.55 6.55
3.62
1.75
-0.38
4.23 2.17 4.06
18.00
10.0 9.5 9.0 8.5 8.0 7.5 7.0 6.5 6.0 5.5 5.0 4.5 4.0 3.5 3.0 2.5 2.0 1.5 1.0 0.5 0.0 -0.5
f1 (ppm)

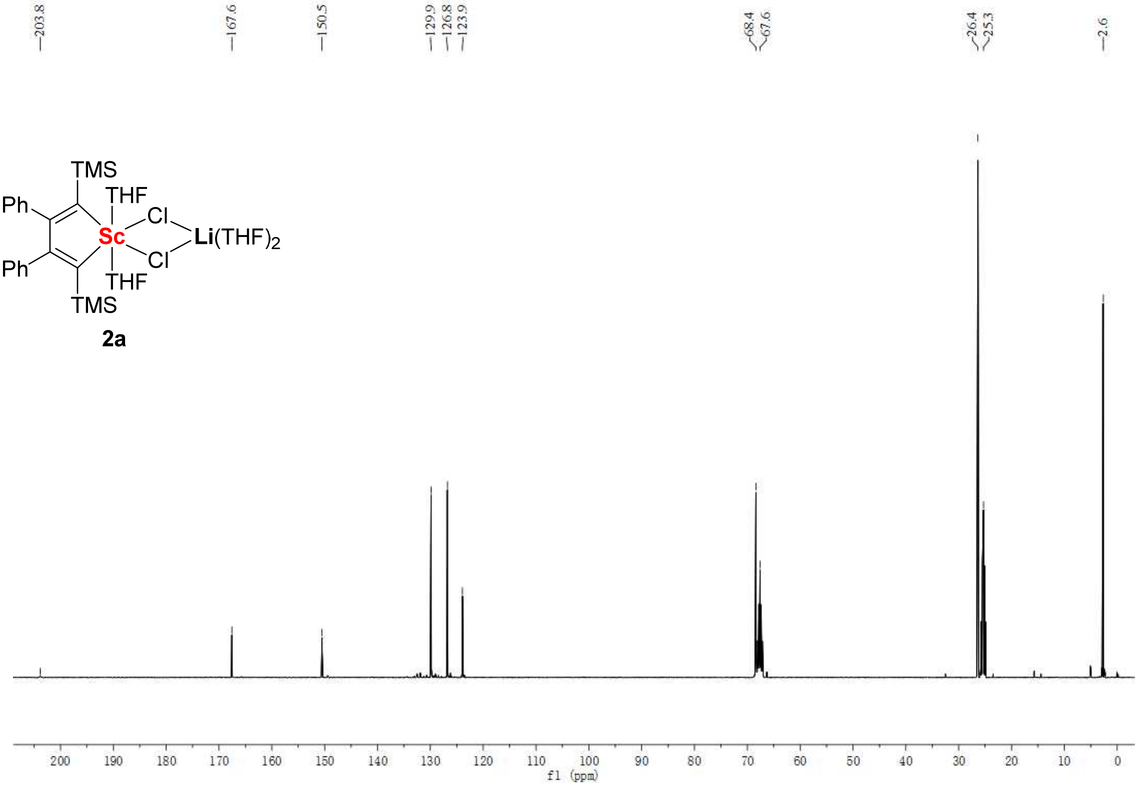
203.8
167.6
150.5
129.9 126.8 123.9
68.4 67.6
26.4 25.3
2.6
TMS
THF
Ph
Cl
Sc
Li(THF)2
Ph
Cl
THF
TMS
2a
200 190 180 170 160 150 140 130 120 110 100 90 80 70 60 50 40 30 20 10 0
f1 (ppm)

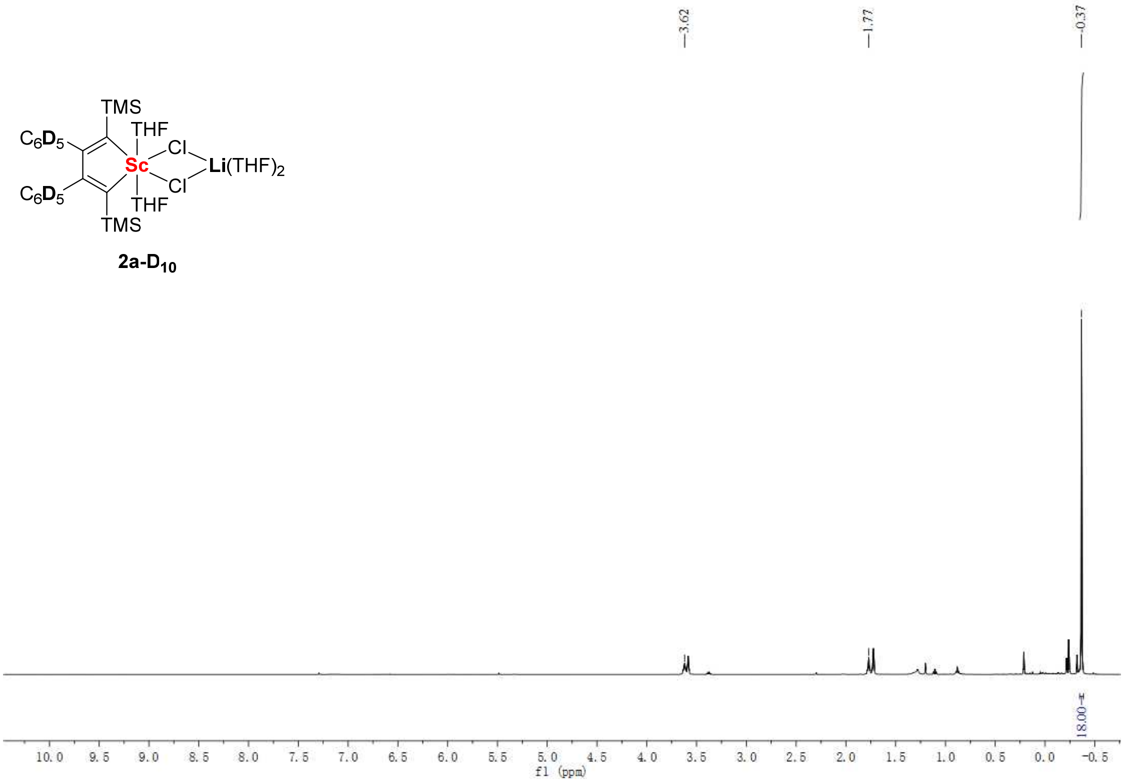
TMS
THF
C_6D_5
Sc
Cl
Li(THF)$_2$
Cl
C_6D_5
THF
TMS
2a-D$_{10}$
3.62
1.77
-0.37
18.00
10.0 9.5 9.0 8.5 8.0 7.5 7.0 6.5 6.0 5.5 5.0 4.5 4.0 3.5 3.0 2.5 2.0 1.5 1.0 0.5 0.0 -0.5
f1 (ppm)

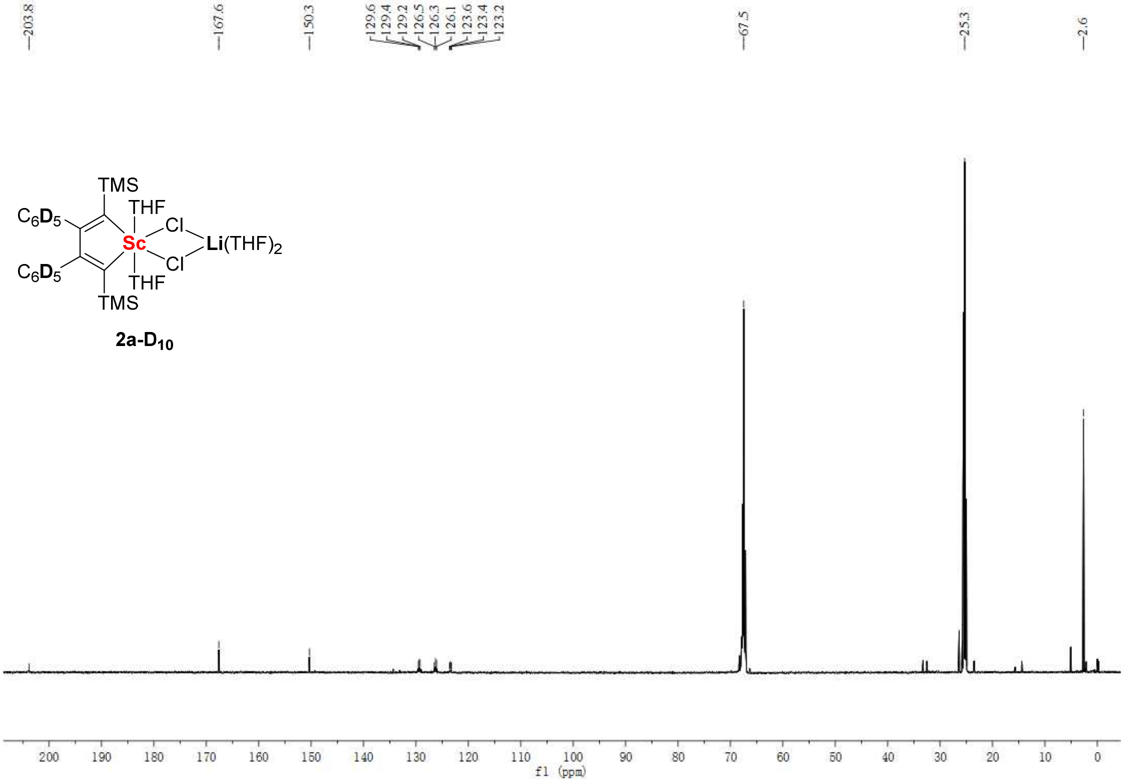
TMS
THF
C_6D_5
Sc
Cl
Li(THF)$_2$
Cl
C_6D_5
THF
TMS
2a-D$_{10}$
203.8
167.6
150.3
129.6
129.4
129.2
126.5
126.3
126.1
123.6
123.4
123.2
67.5
25.3
2.6
200 190 180 170 160 150 140 130 120 110 100 90 80 70 60 50 40 30 20 10 0
f1 (ppm)

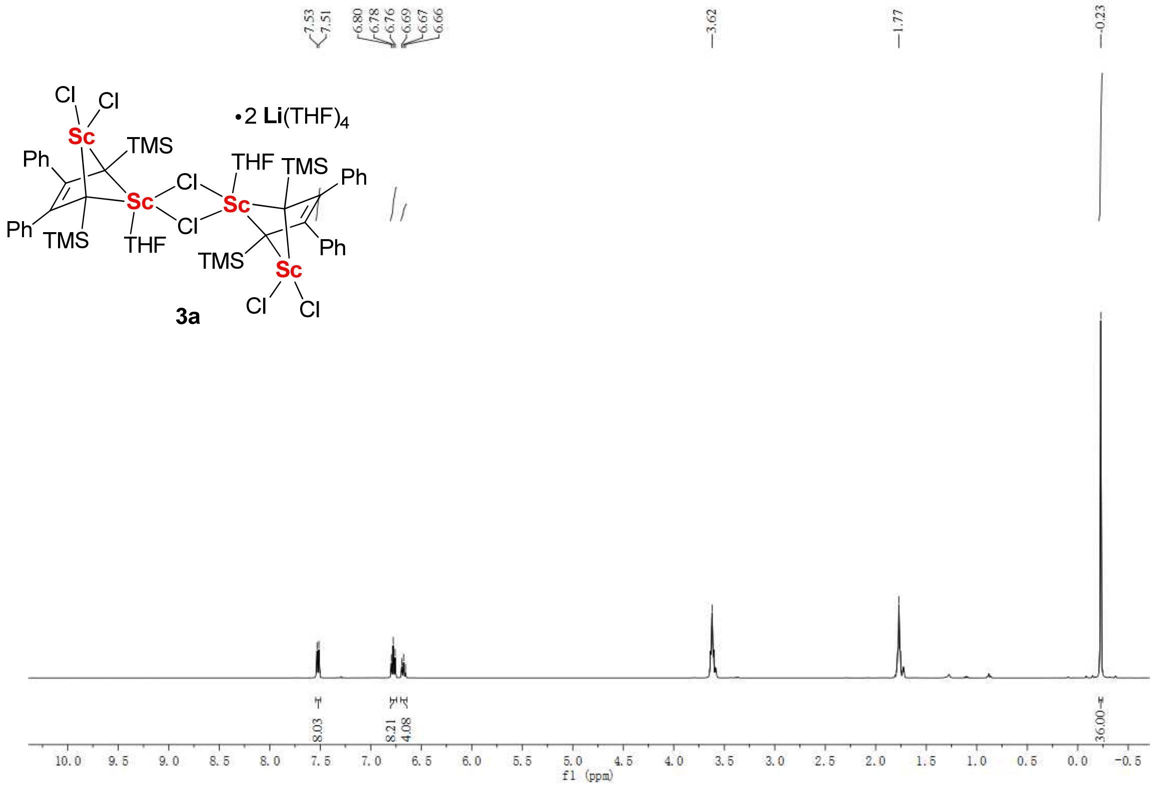
7.53
7.51
6.80
6.78
6.76
6.69
6.67
6.66
3.62
1.77
-0.23
•2 Li(THF)4
3a
8.03
8.21
4.08
36.00
f1 (ppm)

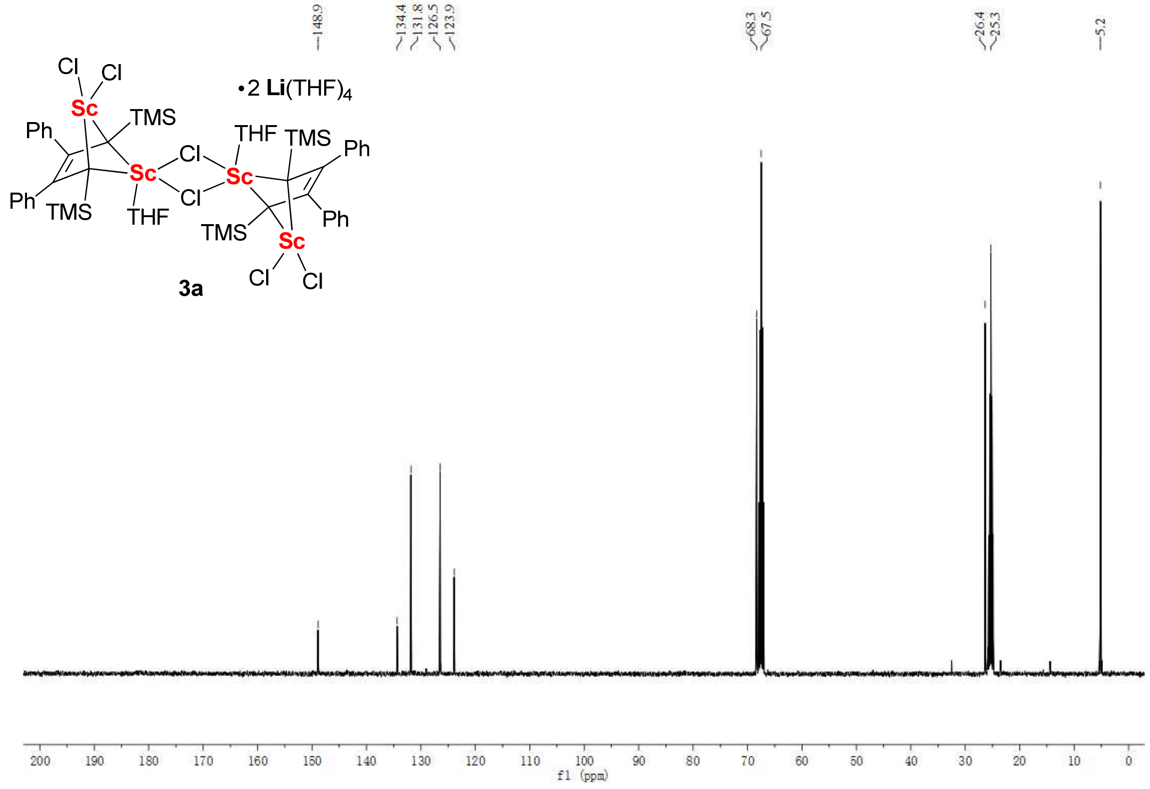
148.9
134.4
131.8
126.5
123.9
68.3
67.5
26.4
25.3
5.2
•2 Li(THF)4
3a
f1 (ppm)

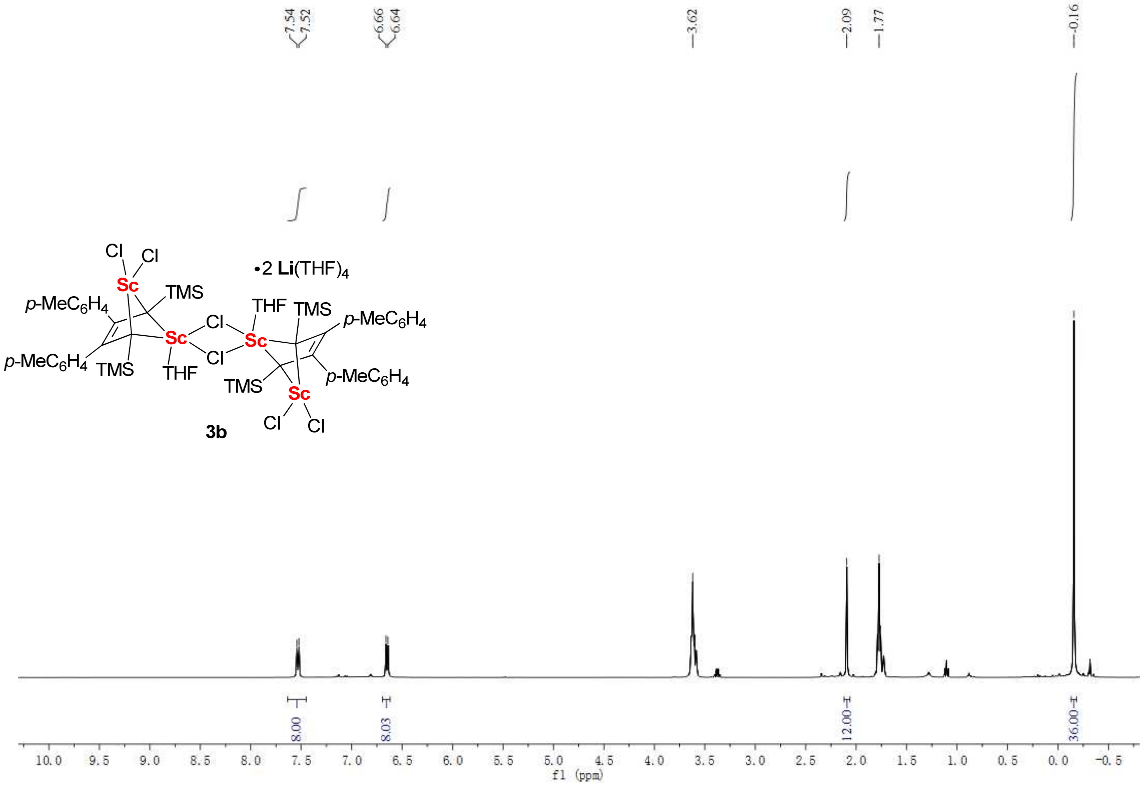
7.54
7.52
6.66
6.64
3.62
2.09
1.77
-0.16
•2 Li(THF)4
3b
8.00
8.03
12.00
36.00
f1 (ppm)

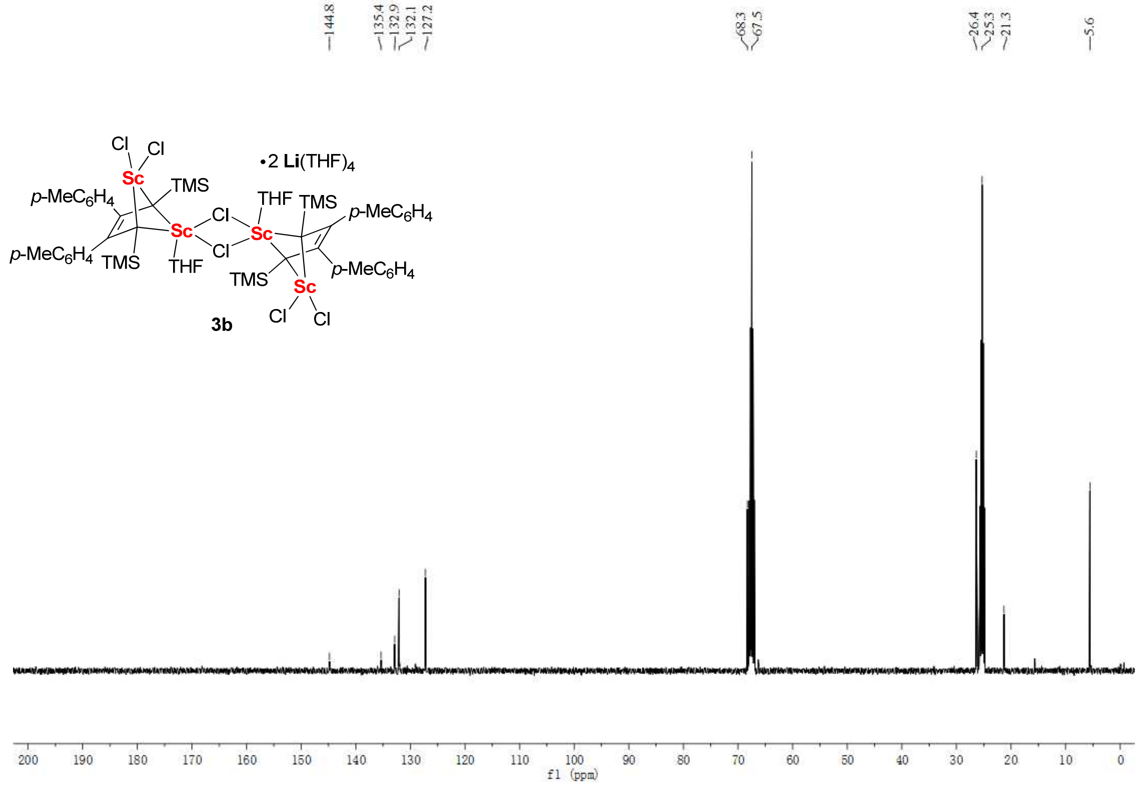
144.8
135.4
132.9
132.1
127.2
68.3
67.5
26.4
25.3
21.3
5.6
•2 Li(THF)4
3b
f1 (ppm)

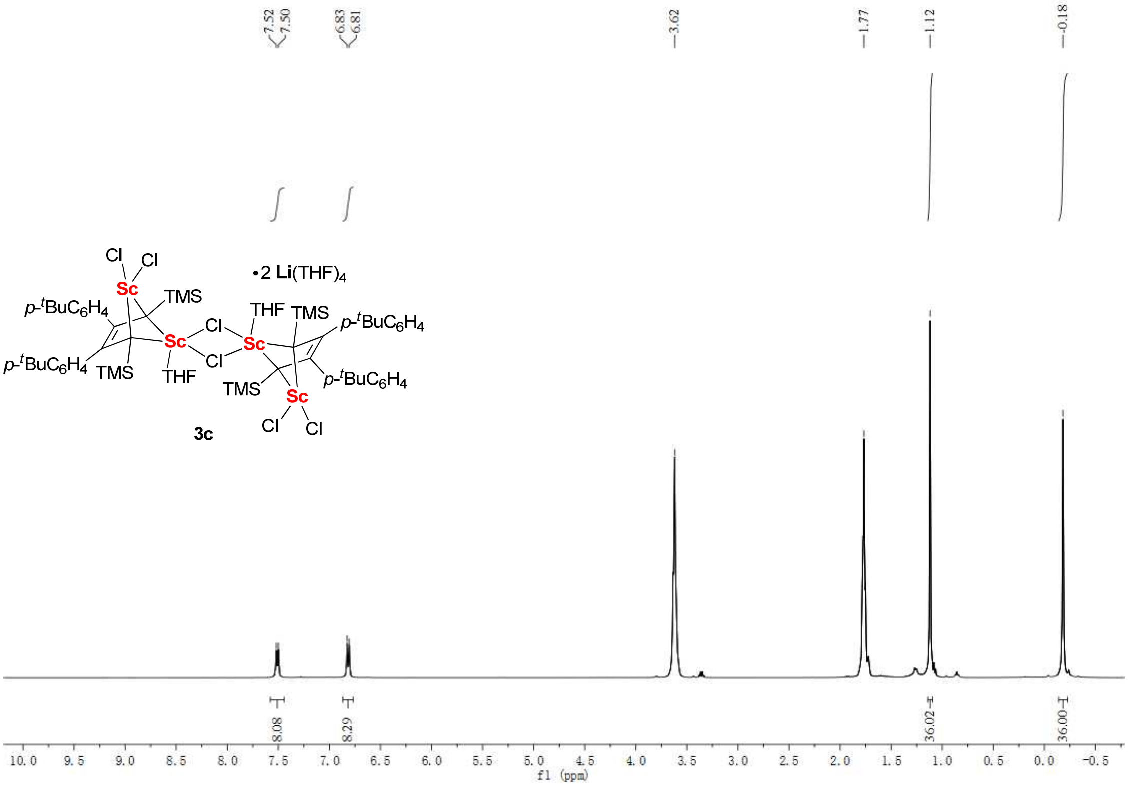

7.52
7.50
6.83
6.81
3.62
1.77
1.12
0.18
Cl
Cl
Sc
TMS
p-^{t}BuC$_6$H$_4$
p-^{t}BuC$_6$H$_4$
TMS
Sc
THF
Cl
Cl
Sc
THF
TMS
p-^{t}BuC$_6$H$_4$
TMS
p-^{t}BuC$_6$H$_4$
Sc
Cl
Cl
•2 Li(THF)$_4$
3c
8.08
8.29
36.02
36.00
10.0 9.5 9.0 8.5 8.0 7.5 7.0 6.5 6.0 5.5 5.0 4.5 4.0 3.5 3.0 2.5 2.0 1.5 1.0 0.5 0.0 -0.5
f1 (ppm)

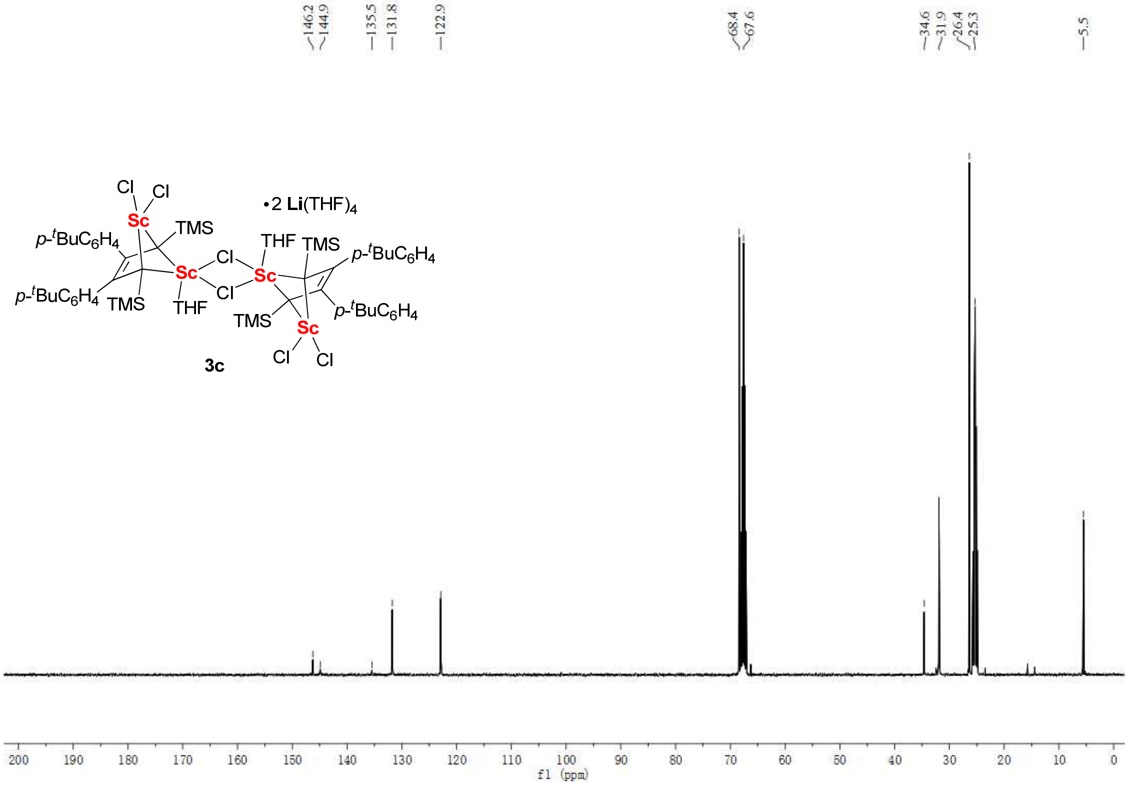

146.2
144.9
135.5
131.8
122.9
68.4
67.6
34.6
31.9
26.4
25.3
5.5
Cl
Cl
Sc
TMS
p-^{t}BuC$_6$H$_4$
p-^{t}BuC$_6$H$_4$
TMS
Sc
THF
Cl
Cl
Sc
THF
TMS
p-^{t}BuC$_6$H$_4$
TMS
p-^{t}BuC$_6$H$_4$
Sc
Cl
Cl
•2 Li(THF)$_4$
3c
200 190 180 170 160 150 140 130 120 110 100 90 80 70 60 50 40 30 20 10 0
f1 (ppm)

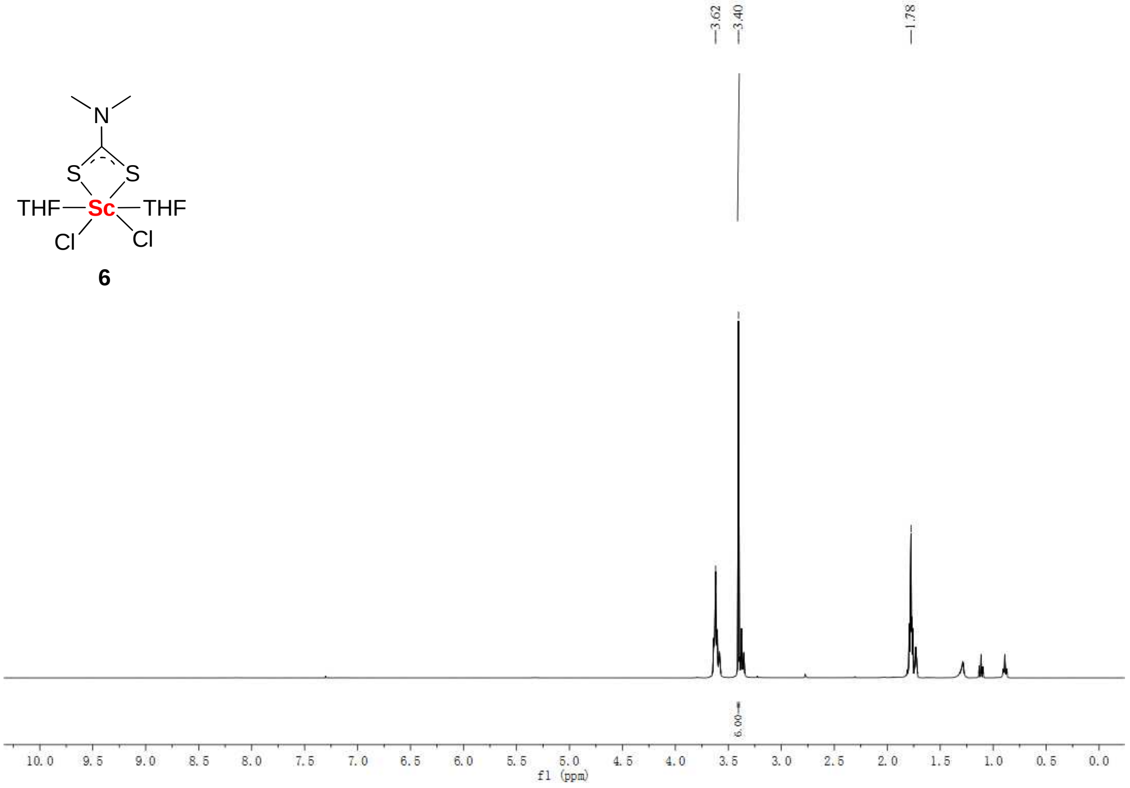
3.62
3.40
1.78
6.00
10.0 9.5 9.0 8.5 8.0 7.5 7.0 6.5 6.0 5.5 5.0 4.5 4.0 3.5 3.0 2.5 2.0 1.5 1.0 0.5 0.0
f1 (ppm)
N
S
S
THF
Sc
THF
Cl
Cl
6

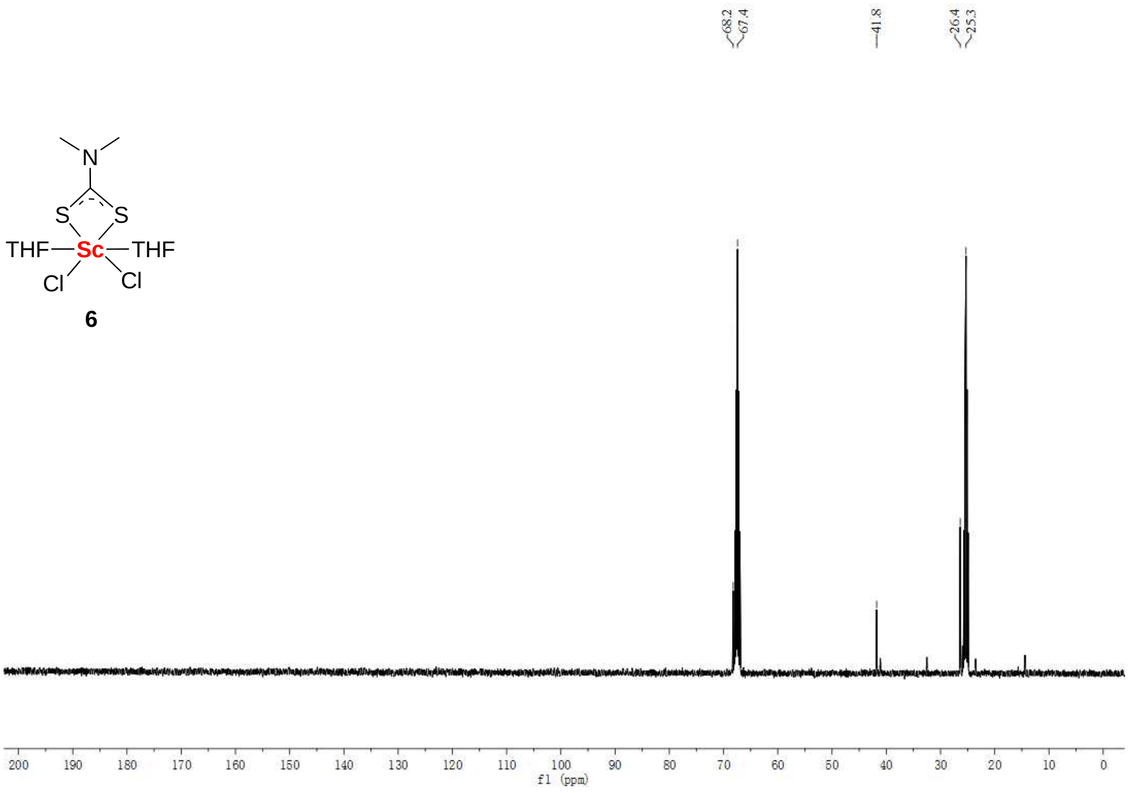
68.2
67.4
41.8
26.4
25.3
200 190 180 170 160 150 140 130 120 110 100 90 80 70 60 50 40 30 20 10 0
f1 (ppm)
N
S
S
THF
Sc
THF
Cl
Cl
6

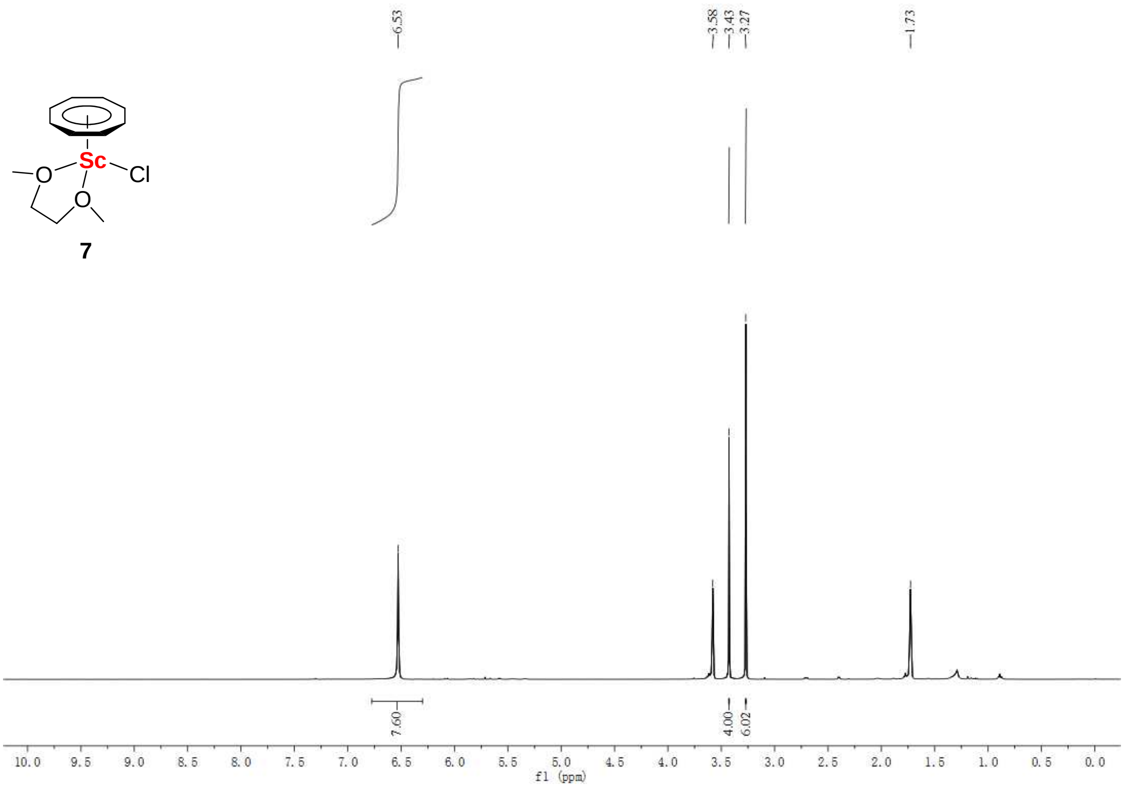
6.53
3.58
3.43
3.27
1.73
Sc
Cl
O
O
7
7.60
4.00
6.02
10.0 9.5 9.0 8.5 8.0 7.5 7.0 6.5 6.0 5.5 5.0 4.5 4.0 3.5 3.0 2.5 2.0 1.5 1.0 0.5 0.0
f1 (ppm)

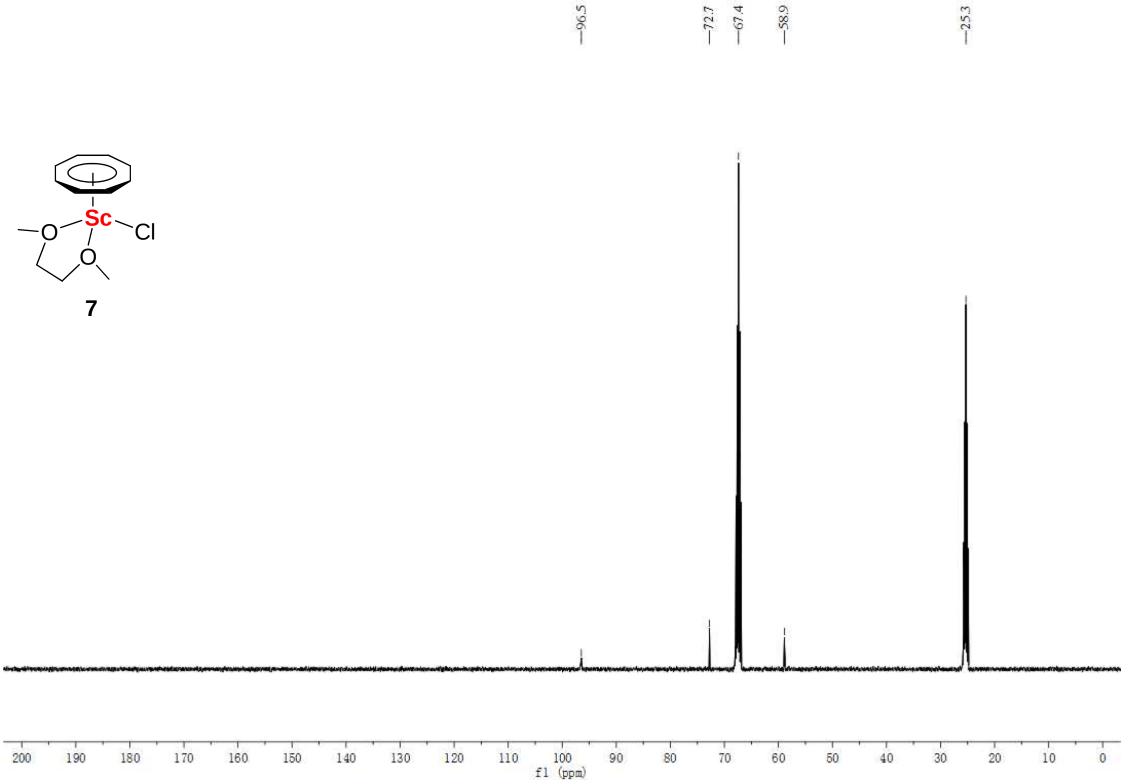
96.5
72.7
67.4
58.9
25.3
Sc
Cl
O
O
7
200 190 180 170 160 150 140 130 120 110 100 90 80 70 60 50 40 30 20 10 0
f1 (ppm)

3) X-ray Crystallographic Studies

The single crystals of **2a**, **3a, 6** and **7** suitable for X-ray analysis were grown as described in experimental section. The crystals were wrapped in mineral oil and then were frozen in low temperature. Data collections were performed on a SuperNova diffractometer, using graphite-monochromated Mo Kα radiation (λ = 0.71073 Å). The structures were solved with the shelxs-97[3] or Olex2[4] and refined with the XL refinement package using Least Squares minimization. Refinement was performed on F^2 anisotropically for all the non-hydrogen atoms by the full-matrix least-squares method. The hydrogen atoms were placed at the calculated positions and were included in the structure calculation without further refinement of the parameters. Disordered solvent in **2a** was squeezed by using Platon.[5] Crystal data, data collection and processing parameters for **2a**, **3a, 6** and **7** were summarized. Crystallographic data have been deposited with the Cambridge Crystallographic Data Centre as supplementary publication nos. CCDC 1555495 (**2a**), CCDC 1504242 (**3a**), 1519012 (**6**), CCDC 1539530 (**7**). Copies of these data can be obtained free of charge from the Cambridge Crystallographic Data Centre via www.ccdc.cam.ac.uk/data_request/cif. The thermal ellipsoid plots were drawn by Ortep-3 v1.08.[6]

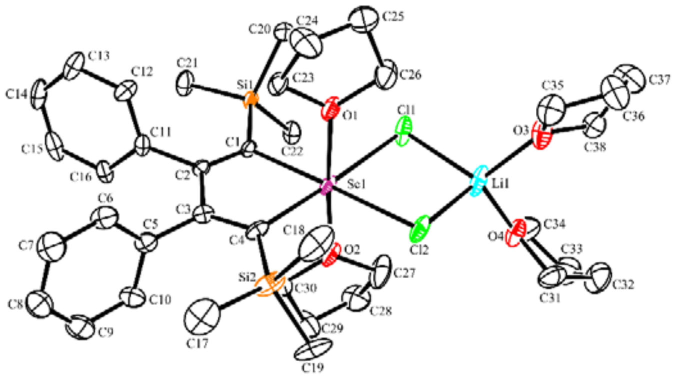

SFigure 1. ORTEP drawing of **2a** with 30% thermal ellipsoids. H atoms are omitted for clarity.

STable 1 Crystal data and structure refinement for 2a.

Identification code	**2a**
Empirical formula	$C_{38}H_{60}Cl_2LiO_4ScSi_2$
Formula weight	759.84
Temperature/K	100.0(2)
Crystal system	triclinic
Space group	P-1
a/Å	12.55479(19)
b/Å	13.3494(4)

c/Å	14.7909(5)
α/°	89.352(3)
β/°	70.920(2)
γ/°	89.305(2)
Volume/Å^3	2342.52(11)
Z	2
ρ_{calc}mg/mm^3	1.077
μ/mm^{-1}	0.354
F(000)	812.0
Crystal size/mm^3	0.15 × 0.10 × 0.10
Radiation	MoKα (λ = 0.71073)
2 Θ range for data collection	7.098 to 52.044°
Index ranges	-17 ⩽ h ⩽ 8, -18 ⩽ k ⩽ 18, -20 ⩽ l ⩽ 20
Reflections collected	67633
Independent reflections	7818 [R_{int} = 0.0476, R_{sigma} = 0.0218]
Data/restraints/parameters	7818/5/439
Goodness-of-fit on F^2	1.163
Final R indexes [I>=2σ (I)]	R_1 = 0.0849, wR_2 = 0.2402
Final R indexes [all data]	R_1 = 0.0915, wR_2 = 0.2498
Largest diff. peak/hole / e Å^{-3}	1.02/-0.80

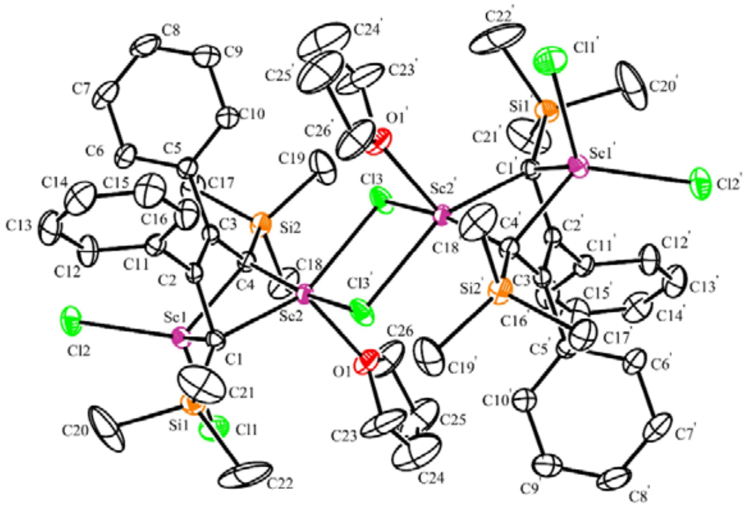

SFigure 2. ORTEP drawing of **3a** with 30% thermal ellipsoids. H atoms, and two $[Li(THF)_4]^+$ counterions are omitted for clarity.

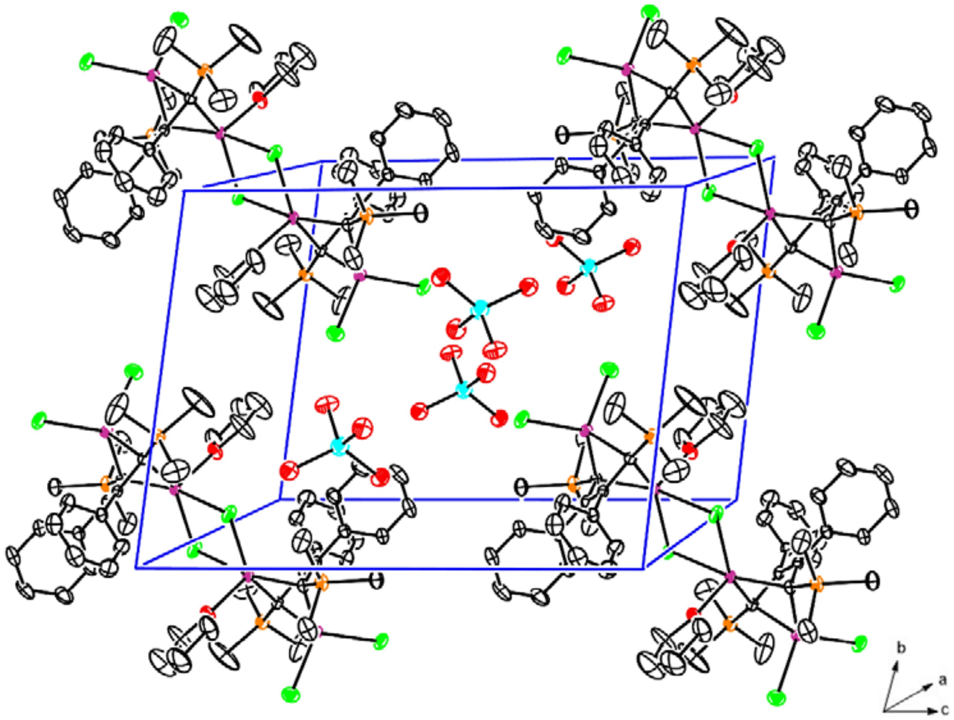

SFigure 3. ORTEP packing drawing of **3a** with 30% thermal ellipsoids. H atoms, and C atoms of THF molecules of $[Li(THF)_4]^+$ counterions are omitted for clarity. Colour code: Sc: violet red, C: black, Li: cyan, O: red, Cl: green, Si: orange. The *a* axis is across through the center of the μ_2-chloride bridges plane.

STable 2 Crystal data and structure refinement for 3a.

Identification code	**3a**
Empirical formula	$C_{84}H_{136}Cl_6Li_2O_{10}Sc_4Si_4$
Formula weight	1824.70
Temperature/K	180.00(10)
Crystal system	triclinic
Space group	P-1
a/Å	12.1887(6)
b/Å	12.7433(7)
c/Å	16.8304(10)
α/°	83.560(5)
β/°	75.676(5)
γ/°	74.313(5)
Volume/Å^3	2435.7(2)
Z	1
ρ_{calc}mg/mm^3	1.244
μ/mm^{-1}	0.531

F(000)	968.0
Crystal size/mm^3	0.05 × 0.05 × 0.05
Radiation	MoKα (λ = 0.71073)
2 Θ range for data collection	5.722 to 52.044°
Index ranges	-14 ⩽ h ⩽ 15, -15 ⩽ k ⩽ 14, -13 ⩽ l ⩽ 20
Reflections collected	16545
Independent reflections	9579 [R_{int} = 0.0306, R_{sigma} = 0.0581]
Data/restraints/parameters	9579/36/502
Goodness-of-fit on F^2	1.035
Final R indexes [I>=2σ (I)]	R_1 = 0.0609, wR_2 = 0.1443
Final R indexes [all data]	R_1 = 0.0888, wR_2 = 0.1668
Largest diff. peak/hole / e Å^{-3}	1.08/-0.54

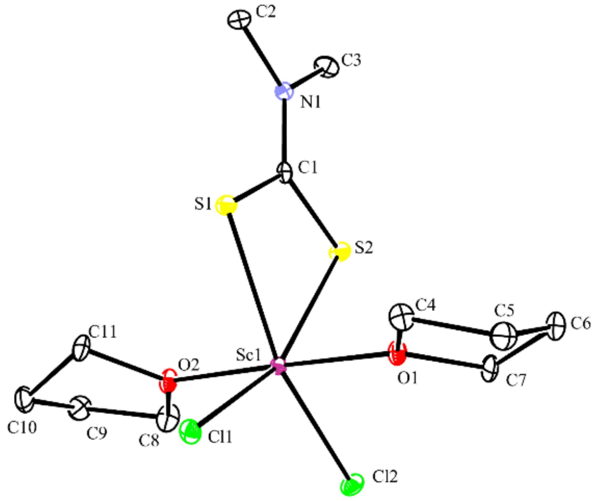

SFigure 4. ORTEP drawing of **6** with 30% thermal ellipsoids. H atoms are omitted for clarity.

STable 3 Crystal data and structure refinement for 6.

Identification code	**6**
Empirical formula	$C_{11}H_{22}Cl_2NO_2S_2Sc$
Formula weight	380.27
Temperature/K	100.00(10)
Crystal system	monoclinic
Space group	$P2_1/n$
a/Å	7.6252(3)
b/Å	17.9614(5)
c/Å	12.5733(4)
α/°	90
β/°	101.302(3)

γ/°	90
Volume/Å^3	1688.64(9)
Z	4
ρ_{calc}mg/mm^3	1.496
μ/mm^{-1}	0.996
F(000)	792.0
Crystal size/mm^3	0.1 × 0.05 × 0.05
Radiation	MoKα (λ = 0.71073)
2 Θ range for data collection	5.612 to 52.04°
Index ranges	-9 ⩽ h ⩽ 9, -22 ⩽ k ⩽ 22, -15 ⩽ l ⩽ 15
Reflections collected	21950
Independent reflections	3333 [R_{int} = 0.0422, R_{sigma} = 0.0242]
Data/restraints/parameters	3336/0/174
Goodness-of-fit on F^2	1.031
Final R indexes [I>=2σ (I)]	R_1 = 0.0250, wR_2 = 0.0597
Final R indexes [all data]	R_1 = 0.0333, wR_2 = 0.0649
Largest diff. peak/hole / e Å^{-3}	0.31/-0.29

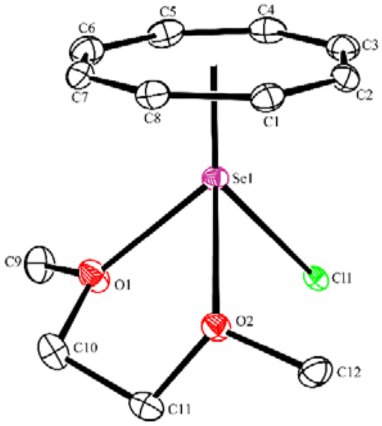

SFigure 5. ORTEP drawing of **7** with 30% thermal ellipsoids. H atoms are omitted for clarity.

STable 3 Crystal data and structure refinement for 7.

Identification code	**7**
Empirical formula	$C_{12}H_{18}ClO_2Sc$
Formula weight	274.67
Temperature/K	100.00(10)
Crystal system	monoclinic
Space group	Pn
a/Å	7.0301(3)
b/Å	10.1171(5)

c/Å	9.2007(4)
α/°	90
β/°	101.433(4)
γ/°	90
Volume/Å^3	641.41(5)
Z	2
ρ_{calc}mg/mm^3	1.422
μ/mm^{-1}	0.765
F(000)	288.0
Crystal size/mm^3	0.15 × 0.14 × 0.12
Radiation	MoKα (λ = 0.71073)
2 Θ range for data collection	7.812 to 52.034°
Index ranges	-8 ⩽ h ⩽ 8, -12 ⩽ k ⩽ 12, -11 ⩽ l ⩽ 11
Reflections collected	14657
Independent reflections	2510 [R_{int} = 0.0430, R_{sigma} = 0.0288]
Data/restraints/parameters	2510/2/147
Goodness-of-fit on F^2	1.080
Final R indexes [I>=2σ (I)]	R_1 = 0.0400, wR_2 = 0.1167
Final R indexes [all data]	R_1 = 0.0407, wR_2 = 0.1173
Largest diff. peak/hole / e Å^{-3}	0.72/-0.61

4) Mechanistic Investigations for the Formation of 3a

***In situ* ^{1}H NMR Monitoring the Transformation from 2a to 3a and Alkyne.** A J.Young valve NMR tube containing THF-d_8 solution of pure **2a** (38.0 mg, 0.05 mmol) was injected into NMR spectrum spectrometer at 25 °C, and the ^{1}H NMR spectra was recorded (SFigure 6 above). The ^{1}H NMR spectra showed no obvious change for 2 weeks at 25 °C. When the NMR tube was heated over 80 °C for 10 min or 45 °C for 3 h in an oil bath, the peak at -0.38 ppm completely disappeared and two new singlets integrated to the same numbers of protons appeared at -0.23 ppm and 0.20 ppm (SFigure 6 bottom). The singlet at 0.20 ppm was assigned to the TMS proton resonance of Ph-C≡C-TMS by comparing with its standard spectrum. The GC retention time and molecular ion peak (m/z = 174) detected by GC-MS are also consistent with the standard sample of Ph-C≡C-TMS. The other new singlet at -0.23 ppm was assigned to the TMS groups of complex **3a**.

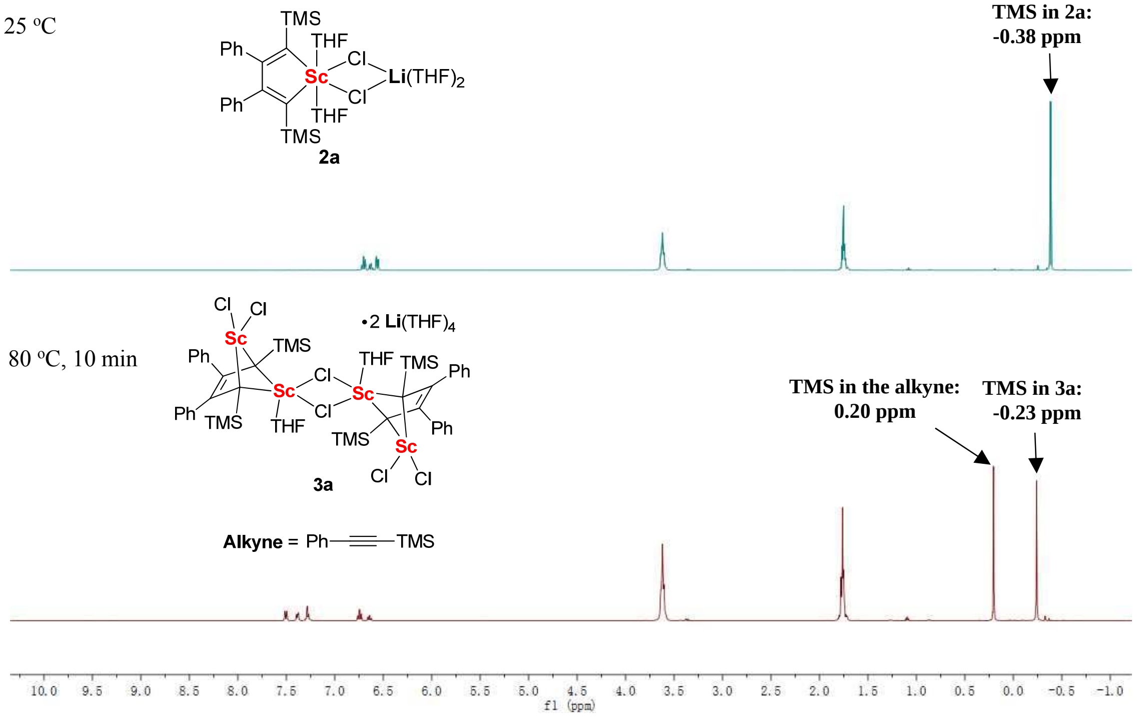

SFigure 6. *In situ* ^{1}H NMR spectra of the transformation from **2a** (above) to **3a** + **alkyne** (bottom). The transformation was observed by the signals of TMS groups of each compound.

***In situ* ^{1}H NMR Monitoring the Transformation from 3a to 2a.** A J.Young valve NMR tube containing THF-d_8 solution of pure **3a** (36.5 mg, 0.02 mmol) with ferrocene as an internal standard was

injected into NMR spectrum spectrometer at 25 ºC, and the ^{1}H NMR spectra was recorded (SFigure 7 above). When 2 equiv of hexachloroethane (9.5 mg, 0.04 mmol) were added into the NMR tube at 25 ºC, the peak at -0.23 ppm completely disappeared and a new singlet appeared at -0.38 ppm which was assigned to the TMS signal of complex **2a** (SFigure 7 bottom). The conversion ratio (70%) was based on the internal standard ferrocene. The formation of $ScCl_3$ can be characterized as $ScCl_3(THF)_3$ determined by X-ray analysis after post processing, cell parameters: a = 8.2062(6)Å, b = 12.5280(9)Å, c = 16.6537Å, α = 90°, β = 93.040(8)°, γ = 90°, its crystal structure have been already reported[7] and thus its crystal structure is not illustrated. The formation of tetrachloroethylene can be identified by the ^{13}C NMR spectrum and GC-MS.

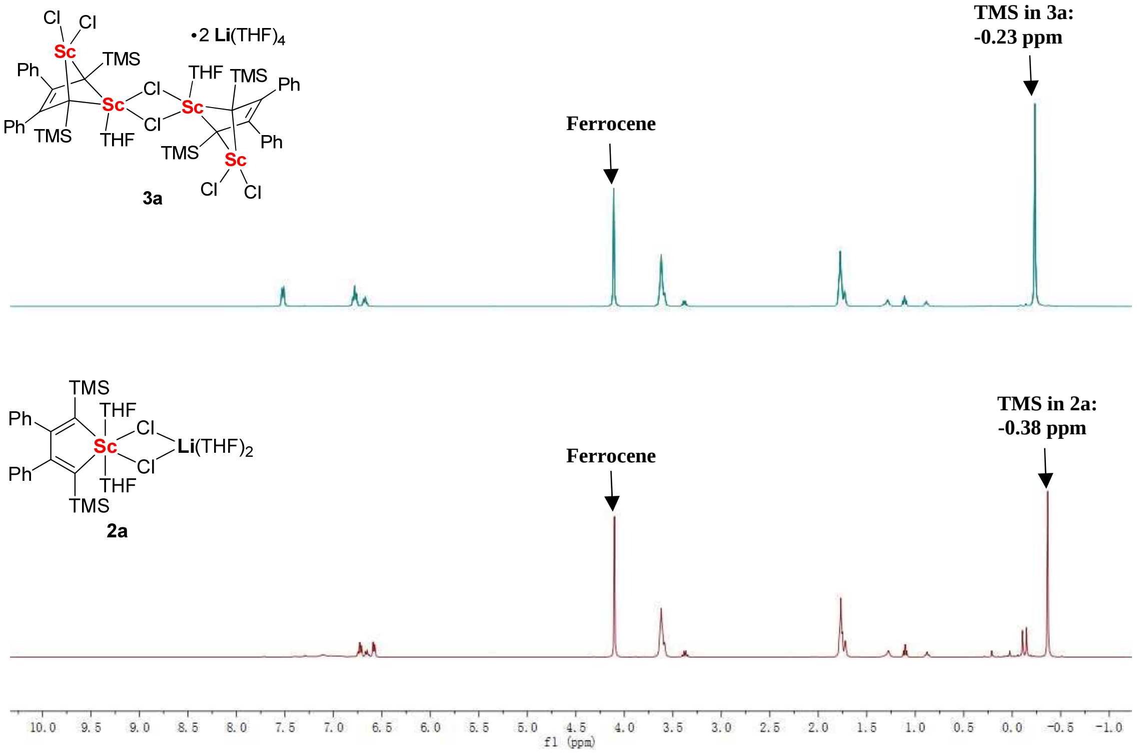

SFigure 7. *In situ* ^{1}H NMR spectra of the transformation from **3a** to **2a** by adding hexachloroethane. The conversion ratio was based on the internal standard ferrocene.

In situ ^{1}**H NMR Monitoring the Transformation from 3a to Alkyne.** A J.Young valve NMR tube containing THF-d_8 solution of pure **3a** (36.5 mg, 0.02 mmol) with ferrocene as an internal standard was injected into NMR spectrum spectrometer at 25 ºC, and the ^{1}H NMR spectra was recorded (SFigure 8 above). When 4 equiv of hexachloroethane (19.0 mg, 0.08 mmol) were added into the NMR tube at 25 ºC, and then the NMR tube was heated over 80 ºC for 12 h in an oil bath. The peak at -0.23 ppm

completely disappeared and a new singlet appeared at 0.20 ppm which was assigned to the TMS signal of Ph-C≡C-TMS (SFigure 8 bottom). The conversion ratio (48%) was based on the internal standard ferrocene. The formation of $ScCl_3$ can be characterized as $ScCl_3(THF)_3$. The formation of tetrachloroethylene can be identified by the ^{13}C NMR spectrum and GC-MS.

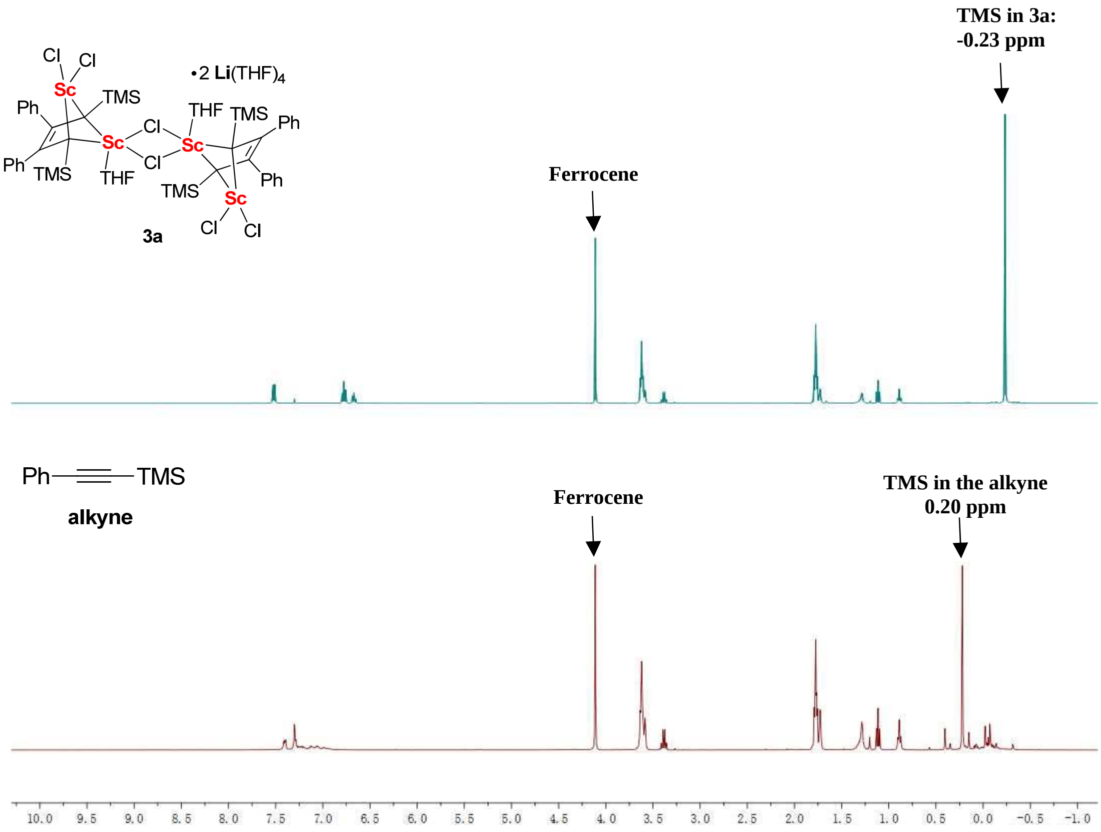

SFigure 8. *In situ* ^1H NMR spectra of the transformation from **3a** to **Ph-C≡C-TMS** by adding hexachloroethane. The conversion ratio was based on the internal standard ferrocene.

The Crossover Reaction among 2a and 2a-D$_{10}$. In the glovebox, **2a** (38.0 mg, 0.05 mmol) and deuterated **2a-D$_{10}$** (38.5 mg, 0.05 mmol) were dissolved and mixed in THF (10 mL) in a 25 mL sealed tube. And then the sealed tube was transferred outside of the glovebox and heated over 80 °C for 3 h in an oil bath. After the heating, the solution was cooled to the room temperature and quenched with excess H_2O. The solution was extracted with petroleum ether and dried over Na_2SO_4. The extract was directly used for high-resolution mass spectra (HRMS) on FTMS mass spectrometer using an EI source. The quenched product **4a**, **4a-D$_5$**, and **4a-D$_{10}$** could all be detected by HRMS (SScheme 1 and SFigure 9). **4a:** HRMS (EI, m/z) calcd for $C_{22}H_{32}Si_2$ $[M]^+$: 352.2037, found 352.2037; **4a-D$_5$:** HRMS (EI, m/z) calcd for

$C_{22}H_{27}D_5Si_2$ $[M]^+$: 357.2351, found 357.2352; **4a-D$_{10}$:** HRMS (EI, m/z) calcd for $C_{22}H_{22}D_{10}Si_2$ $[M]^+$: 362.2665, found 362.2667. The detection of the crossover product **4a-D$_5$** by HRMS revealed that the 2-butene-1,1,4,4-tetraanion moiety in **3a** should be originated from two molecules of scandacyclopentadienes instead of a simple reduction of a diene moiety in one scandacyclopentadiene.

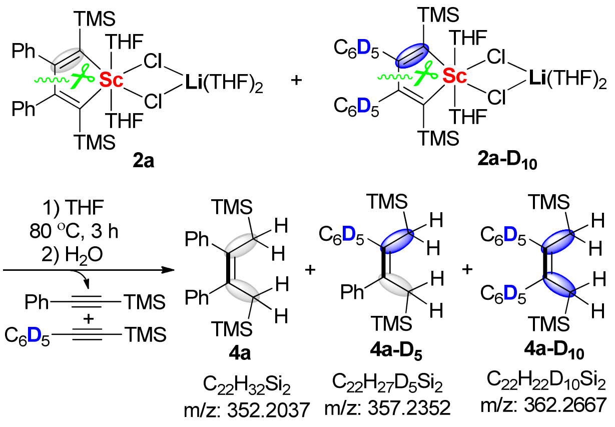

SScheme 1. The crossover reaction among **2a** and **2a-D$_{10}$**.

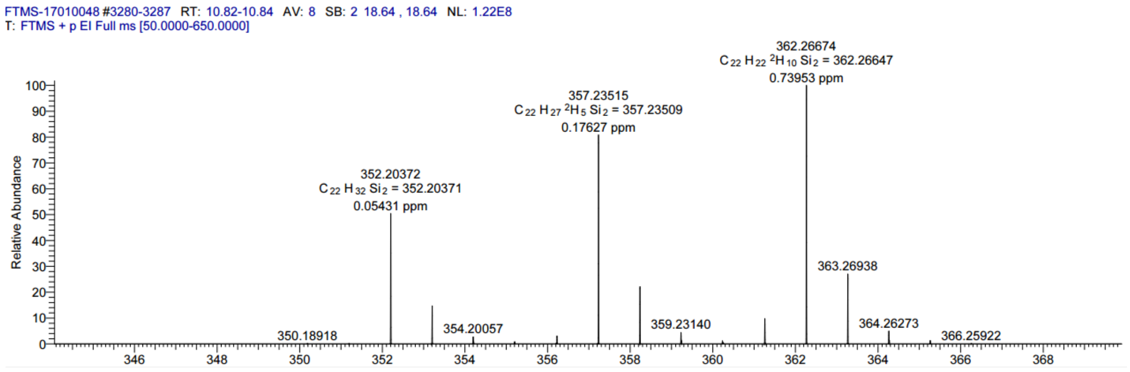

SFigure 9. HRMS spectra of the quenched products of the crossover reaction.

Proposed Mechanisms for the Formation of 3a from 2a. Three possible mechanism pathways are illustrated in the **SScheme 2**. *Pathway (**a**)*: cooperative intermolecular redox process. One molecule of scandacyclopendadiene **2a** transfers its "ScCl" moiety to another molecule of **2a** to generate **3a** along with cleavage of β,β'-C−C bond of one molecule of scandacyclopendadiene to eliminate two equiv of alkyne; *Pathway (**b**)*: stepwise intermolecular redox via scandacyclopropene. One molecule of **2a** undergoes β,β'-C−C bond cleavage to generate the scandacyclopropene along with the elimination of

one equiv of alkyne, and then the scandacyclopropene transfers its “ScCl” moiety to another molecule of **2a** to generate **3a**. *Pathway (c)*: dimerization of scandacyclopropene via cooperative double metathesis. Two molecules of **2a** will undergo *β*,*β*′-C−C bond cleavage to generate scandacyclopropene by release of two equiv of alkyne, and then two molecules of scandacyclopropene undergo dimerization to generate **3a**.

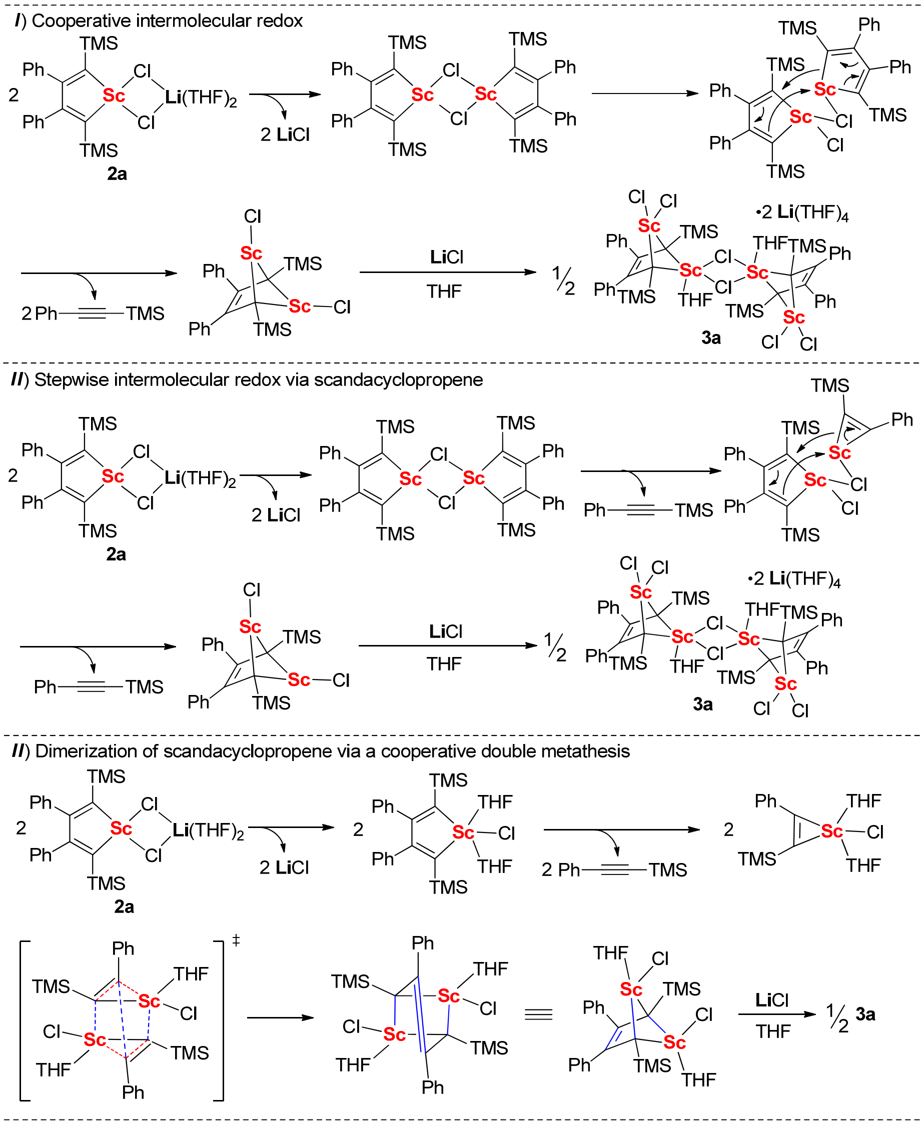

SScheme 2. Proposed mechanisms for the formation of **3a** from **2a**.

5) Details of DFT Calculations

All calculations were carried out with the GAUSSIAN 09 program package.[8] The optimization structure and correction energy of all the minima and transition states were fully calculated at the B3LYP level[9] using the LANL2DZ (for Sc) and 6-31+G(d) (for other elements) in gas phase. The effect of solvent was examined by performing single-point self-consistent reaction field (SCRF) calculations based on the polarizable continuum model (PCM) for gas-phase optimized structures. THF was used as solvent, and solvation free energies (ΔG_{sol}) were calculated at M06/SDD (for Sc)/ 6-311+G(d,p) (for other elements) level, by adding the solvation energies to the computed gas phase relative free energies (ΔG_{gas}). Harmonic frequency calculations were performed at the same level for every structure to confirm it as a local minimum or transition state and to derive the thermochemical corrections for enthalpies and free energies. The intrinsic reaction coordinate (IRC) analysis was carried out throughout the pathways to confirm that all stationary points are smoothly connected to each other. The NBO analysis[10] at B3LYP/SDD (for Sc)/ 6-311+G(d,p) (for other elements) level. All enthalpies and the Gibbs free energies in the text were given in Hartree. All distances were given in Å.

IM1
298K, 1 atm, gas phase
Thermal correction to Enthalpy = 0.714314 Hartree
Thermal correction to Gibbs Free Energy = 0.584531 Hartree
Sum of electronic and thermal Enthalpies = -2405.245457 Hartree
Sum of electronic and thermal Free Energies = -2405.375240 Hartree
298K, in THF
Sum of electronic and thermal Free Energies= 2404.997502 Hartree

Standard orientation:

Center Number	Atomic Number	Atomic Type	Coordinates (Angstroms) X	Y	Z
1	6	0	0.192822	1.492350	0.204344
2	6	0	-0.969422	0.784869	0.031567
3	6	0	-1.013018	-0.736917	-0.139251
4	6	0	0.108827	-1.503735	-0.320727
5	21	0	1.695318	-0.076377	0.135948
6	14	0	0.247522	-3.345682	-0.636895
7	14	0	0.457251	3.348441	0.236476
8	6	0	2.046611	-3.869034	-0.290302
9	1	0	2.205953	-4.911124	-0.599194

10	1	0	2.774217	-3.252569	-0.834497
11	1	0	2.303410	-3.809446	0.774763
12	6	0	-0.058201	-3.742289	-2.477942
13	1	0	0.200784	-4.788844	-2.688837
14	1	0	-1.106131	-3.596646	-2.764307
15	1	0	0.560082	-3.113728	-3.132647
16	6	0	-0.859911	-4.472715	0.423354
17	1	0	-0.625662	-5.527489	0.222328
18	1	0	-0.697030	-4.298198	1.495042
19	1	0	-1.925688	-4.318252	0.224359
20	6	0	0.322938	4.080168	1.991862
21	1	0	1.042054	3.622651	2.682754
22	1	0	0.539294	5.156844	1.964184
23	1	0	-0.680957	3.956202	2.415231
24	6	0	2.246559	3.661399	-0.340916
25	1	0	2.396115	3.334128	-1.378654
26	1	0	2.486420	4.732518	-0.300695
27	1	0	2.986112	3.135490	0.277317
28	6	0	-0.659400	4.372228	-0.915509
29	1	0	-0.319068	5.417248	-0.926062
30	1	0	-0.618592	4.003001	-1.948476
31	1	0	-1.709509	4.361913	-0.605481
32	6	0	-2.383350	-1.365979	-0.068370
33	6	0	-3.043312	-1.491749	1.165055
34	6	0	-3.014279	-1.882422	-1.210244
35	6	0	-4.281524	-2.130999	1.257791
36	1	0	-2.574766	-1.092522	2.061632
37	6	0	-4.257303	-2.515868	-1.124776
38	1	0	-2.527415	-1.781704	-2.176719
39	6	0	-4.895502	-2.646966	0.111857
40	1	0	-4.768561	-2.225130	2.225727
41	1	0	-4.727130	-2.905381	-2.025094
42	1	0	-5.860949	-3.141633	0.181986
43	6	0	-2.299653	1.493277	-0.045961
44	6	0	-3.005876	1.590256	-1.255918
45	6	0	-2.841686	2.120296	1.086684
46	6	0	-4.200633	2.308351	-1.337176
47	1	0	-2.609619	1.106798	-2.145597
48	6	0	-4.043588	2.829925	1.014568
49	1	0	-2.314733	2.045446	2.034567
50	6	0	-4.726429	2.931686	-0.200753
51	1	0	-4.723072	2.379967	-2.288349
52	1	0	-4.445168	3.303647	1.907461
53	1	0	-5.658917	3.487070	-0.261861
54	17	0	4.110999	-0.156498	0.580507
55	8	0	2.184320	0.296321	-2.019966

56	6	0	3.508842	0.464462	-2.631693
57	6	0	1.142023	0.440122	-3.032628
58	6	0	3.233317	0.921707	-4.063571
59	1	0	4.067209	1.178466	-2.026085
60	1	0	4.013452	-0.504812	-2.587672
61	6	0	1.865990	0.288432	-4.366749
62	1	0	0.387780	-0.321954	-2.830442
63	1	0	0.694499	1.433234	-2.916380
64	1	0	4.021128	0.597928	-4.750955
65	1	0	3.166880	2.015149	-4.114500
66	1	0	1.978214	-0.771438	-4.625664
67	1	0	1.331264	0.784792	-5.182785
68	8	0	1.478100	-0.465892	2.353175
69	6	0	1.960825	-1.703739	2.963113
70	6	0	1.456503	0.601971	3.341266
71	6	0	2.300947	-1.347585	4.414017
72	1	0	1.147935	-2.432510	2.883110
73	1	0	2.827907	-2.045083	2.392871
74	6	0	1.418102	-0.117759	4.684987
75	1	0	2.369327	1.200118	3.225902
76	1	0	0.582656	1.217488	3.125941
77	1	0	3.360497	-1.082086	4.500362
78	1	0	2.100905	-2.179581	5.096290
79	1	0	1.794513	0.509159	5.499394
80	1	0	0.392193	-0.419258	4.928538

Trimethylsilylphenylacetylene
298K, 1 atm, gas phase
Thermal correction to Enthalpy = 0.227889 Hartree
Thermal correction to Gibbs Free Energy = 0.169967 Hartree
Sum of electronic and thermal Enthalpies = -716.883619 Hartree
Sum of electronic and thermal Free Energies = -716.941540 Hartree
298K, in THF
Sum of electronic and thermal Free Energies= -716.6949 Hartree

Standard orientation:

Center Number	Atomic Number	Atomic Type	Coordinates (Angstroms) X	Y	Z
1	6	0	3.877435	1.208669	0.000205
2	6	0	2.483163	1.214727	0.001851
3	6	0	1.765015	0.001506	0.001976
4	6	0	2.480254	-1.213449	0.000686
5	6	0	3.874526	-1.210792	-0.001010

6	6	0	4.578023	-0.001910	-0.001268
7	1	0	4.418799	2.151304	0.000032
8	1	0	1.936947	2.153446	0.002861
9	1	0	1.931787	-2.150854	0.000753
10	1	0	4.413588	-2.154748	-0.002153
11	1	0	5.664930	-0.003224	-0.002624
12	6	0	0.335214	0.002940	0.002852
13	6	0	-0.887644	0.003735	0.002464
14	14	0	-2.731515	0.000444	-0.000010
15	6	0	-3.329001	-1.266503	-1.270716
16	1	0	-2.969358	-2.273570	-1.027073
17	1	0	-4.425857	-1.300345	-1.303371
18	1	0	-2.970169	-1.019181	-2.277078
19	6	0	-3.338240	-0.468867	1.728854
20	1	0	-2.983565	0.244924	2.482094
21	1	0	-4.435380	-0.482288	1.767867
22	1	0	-2.979425	-1.463981	2.018257
23	6	0	-3.334605	1.732034	-0.462710
24	1	0	-2.978388	2.482067	0.253827
25	1	0	-2.975941	2.023907	-1.457156
26	1	0	-4.431590	1.773781	-0.475201

TS1
298K, 1 atm, gas phase
Thermal correction to Enthalpy = 0.712506 Hartree
Thermal correction to Gibbs Free Energy = 0.581805 Hartree
Sum of electronic and thermal Enthalpies = -2405.224408 Hartree
Sum of electronic and thermal Free Energies = -2405.355110 Hartree
298K, in THF
Sum of electronic and thermal Free Energies= -2404.976417 Hartree

Standard orientation:

Center Number	Atomic Number	Atomic Type	Coordinates (Angstroms) X	Y	Z
1	6	0	1.418542	1.199993	0.461957
2	6	0	0.208890	1.378301	0.006524
3	6	0	-1.355438	-0.253441	-0.193733
4	6	0	-1.076377	-1.468839	-0.575987
5	21	0	0.874481	-0.885972	0.209606
6	14	0	-1.948549	-3.025369	-1.102180
7	14	0	2.982821	2.183067	0.693223
8	6	0	-0.957646	-4.549362	-0.566011
9	1	0	-1.378719	-5.458799	-1.015631
10	1	0	0.095044	-4.477791	-0.863442

11	1	0	-0.975522	-4.687033	0.522504
12	6	0	-2.038119	-3.007806	-3.002127
13	1	0	-2.517032	-3.922189	-3.377093
14	1	0	-2.617441	-2.152851	-3.373453
15	1	0	-1.036251	-2.952309	-3.446554
16	6	0	-3.704937	-3.189208	-0.397219
17	1	0	-4.159842	-4.138083	-0.713639
18	1	0	-3.694413	-3.177773	0.700190
19	1	0	-4.360332	-2.374382	-0.724630
20	6	0	3.210145	2.569276	2.539770
21	1	0	3.309402	1.652187	3.132887
22	1	0	4.120028	3.164016	2.696170
23	1	0	2.366381	3.143663	2.943086
24	6	0	4.457034	1.142998	0.109368
25	1	0	4.399475	0.944106	-0.968647
26	1	0	5.403618	1.667433	0.296508
27	1	0	4.497097	0.175371	0.624114
28	6	0	2.986324	3.813194	-0.280966
29	1	0	3.949260	4.329518	-0.164358
30	1	0	2.831389	3.637503	-1.353089
31	1	0	2.195224	4.493883	0.053043
32	6	0	-2.590133	0.474629	0.132838
33	6	0	-2.763142	1.054909	1.401511
34	6	0	-3.659136	0.534154	-0.779095
35	6	0	-3.973117	1.653393	1.756001
36	1	0	-1.937572	1.023670	2.107393
37	6	0	-4.863056	1.149618	-0.432782
38	1	0	-3.536072	0.097901	-1.766968
39	6	0	-5.027798	1.707808	0.839005
40	1	0	-4.090749	2.086066	2.746876
41	1	0	-5.673995	1.192418	-1.156229
42	1	0	-5.966735	2.183422	1.110858
43	6	0	-0.528901	2.551570	-0.487065
44	6	0	-1.033585	2.610800	-1.797737
45	6	0	-0.679885	3.685841	0.332266
46	6	0	-1.639127	3.771199	-2.282762
47	1	0	-0.946014	1.738944	-2.439595
48	6	0	-1.301959	4.840320	-0.144849
49	1	0	-0.305907	3.651958	1.352249
50	6	0	-1.779493	4.891676	-1.458305
51	1	0	-2.010608	3.797179	-3.304596
52	1	0	-1.411796	5.702138	0.509396
53	1	0	-2.259937	5.791923	-1.832853
54	17	0	2.474560	-2.632494	0.893625
55	8	0	1.861752	-0.982826	-1.844303
56	6	0	3.016391	-1.783027	-2.256154

57	6	0	1.306199	-0.273778	-2.988145
58	6	0	3.352310	-1.296045	-3.664280
59	1	0	3.806300	-1.629962	-1.521205
60	1	0	2.716886	-2.835321	-2.234168
61	6	0	1.975817	-0.891744	-4.214632
62	1	0	0.222140	-0.403402	-2.959804
63	1	0	1.547241	0.789204	-2.874407
64	1	0	3.843559	-2.073004	-4.258595
65	1	0	4.019641	-0.426465	-3.623196
66	1	0	1.421336	-1.776493	-4.550045
67	1	0	2.031860	-0.187660	-5.050923
68	8	0	0.200269	-1.052943	2.407302
69	6	0	-0.494995	-2.254853	2.857459
70	6	0	1.011898	-0.515711	3.485732
71	6	0	-0.046626	-2.482079	4.305400
72	1	0	-1.567856	-2.053660	2.769331
73	1	0	-0.216935	-3.078255	2.195802
74	6	0	0.385378	-1.077329	4.758590
75	1	0	2.042919	-0.863094	3.349088
76	1	0	0.976471	0.571703	3.400343
77	1	0	0.806752	-3.168436	4.332816
78	1	0	-0.847178	-2.905671	4.920166
79	1	0	1.092655	-1.095320	5.593886
80	1	0	-0.484104	-0.478782	5.057070

IM2
298K, 1 atm, gas phase
Thermal correction to Enthalpy = 0.483904 Hartree
Thermal correction to Gibbs Free Energy = 0.383249 Hartree
Sum of electronic and thermal Enthalpies = -1688.348108 Hartree
Sum of electronic and thermal Free Energies = -1688.448762 Hartree
298K, in THF
Sum of electronic and thermal Free Energies= -1688.285896 Hartree

Standard orientation:

Center Number	Atomic Number	Atomic Type	Coordinates (Angstroms) X	Y	Z
1	21	0	1.089437	0.012551	0.380337
2	17	0	2.902172	-0.362839	1.986653
3	6	0	-0.955620	0.054695	-0.225233
4	6	0	-0.764315	-0.899907	0.711486
5	14	0	-1.693642	-2.220577	1.633651
6	6	0	-3.361484	-2.729134	0.867093
7	1	0	-3.822700	-3.543967	1.442714

8	1	0	-4.071195	-1.894195	0.836951
9	1	0	-3.233370	-3.083245	-0.164198
10	6	0	-1.990021	-1.625647	3.416082
11	1	0	-2.443583	-2.417083	4.027976
12	1	0	-1.045128	-1.333380	3.891023
13	1	0	-2.660447	-0.756861	3.445355
14	6	0	-0.599824	-3.775179	1.700337
15	1	0	-1.073966	-4.574617	2.285534
16	1	0	-0.410427	-4.171878	0.693511
17	1	0	0.371968	-3.551828	2.158380
18	6	0	-2.170018	0.548534	-0.883914
19	6	0	-3.329451	0.887990	-0.151975
20	6	0	-4.459365	1.415126	-0.780917
21	1	0	-3.330520	0.737163	0.924953
22	6	0	-3.339278	1.270214	-2.913116
23	6	0	-4.476780	1.601292	-2.167637
24	1	0	-5.334730	1.671575	-0.187486
25	1	0	-3.336126	1.412372	-3.991994
26	1	0	-5.359798	2.002551	-2.658679
27	8	0	2.076135	-0.943532	-1.350409
28	6	0	3.482592	-1.373915	-1.361668
29	6	0	1.371095	-1.489814	-2.512284
30	6	0	3.679391	-2.055739	-2.715318
31	1	0	4.101395	-0.487281	-1.209289
32	1	0	3.622902	-2.051689	-0.516015
33	6	0	2.276647	-2.603743	-3.027523
34	1	0	0.386790	-1.812788	-2.171962
35	1	0	1.256171	-0.680298	-3.242217
36	1	0	4.444817	-2.836229	-2.667156
37	1	0	3.983913	-1.327698	-3.476905
38	1	0	2.092027	-3.533154	-2.476168
39	1	0	2.119332	-2.800380	-4.092586
40	6	0	-2.200101	0.773622	-2.277185
41	1	0	-1.310006	0.547894	-2.861471
42	8	0	1.218223	2.237638	0.546611
43	6	0	0.109908	3.173558	0.336005
44	6	0	2.398029	2.954682	1.039098
45	6	0	0.555882	4.473176	1.002372
46	1	0	-0.030117	3.282728	-0.744183
47	1	0	-0.786726	2.724559	0.762764
48	6	0	2.082396	4.432608	0.822242
49	1	0	2.527601	2.697360	2.094115
50	1	0	3.264152	2.591146	0.481840
51	1	0	0.295146	4.468975	2.067510
52	1	0	0.090105	5.349738	0.541318
53	1	0	2.618057	5.072033	1.530850

54	1	0	2.357398	4.743888	-0.193062

THF
298K, 1 atm, gas phase
Thermal correction to Enthalpy = 0.121427 Hartree
Thermal correction to Gibbs Free Energy = 0.090240 Hartree
Sum of electronic and thermal Enthalpies = -232.332920 Hartree
Sum of electronic and thermal Free Energies = -232.364106 Hartree
298K, in THF
Sum of electronic and thermal Free Energies= -232.2554 Hartree

Standard orientation:

Center Number	Atomic Number	Atomic Type	Coordinates (Angstroms) X	Y	Z
1	6	0	1.187429	-0.461502	0.000000
2	8	0	0.000012	-1.258210	-0.000001
3	6	0	-1.187420	-0.461523	0.000000
4	6	0	-0.774622	1.027075	0.000001
5	6	0	0.774604	1.027086	0.000000
6	1	0	1.777788	-0.723826	0.887630
7	1	0	1.777788	-0.723825	-0.887631
8	1	0	-1.777778	-0.723858	-0.887629
9	1	0	-1.777778	-0.723859	0.887628
10	1	0	-1.170597	1.543548	-0.880252
11	1	0	-1.170597	1.543547	0.880254
12	1	0	1.170567	1.543569	-0.880252
13	1	0	1.170567	1.543568	0.880253

IM3
298K, 1 atm, gas phase
Thermal correction to Enthalpy = 0.719475 Hartree
Thermal correction to Gibbs Free Energy = 0.578970 Hartree
Sum of electronic and thermal Enthalpies = -2912.031003 Hartree
Sum of electronic and thermal Free Energies = -2912.171507 Hartree
298K, in THF
Sum of electronic and thermal Free Energies= -2912.069630 Hartree

Standard orientation:

Center Number	Atomic Number	Atomic Type	Coordinates (Angstroms) X	Y	Z
1	21	0	0.320666	1.092053	-0.885919

2	17	0	0.726158	2.452236	-2.810488
3	6	0	1.586102	-0.806652	-0.621535
4	6	0	2.171921	0.329765	-0.175230
5	6	0	-1.742070	0.042741	0.242500
6	6	0	-1.789598	-0.018917	-1.109209
7	21	0	-0.517027	-1.690244	-0.322670
8	14	0	-3.003284	0.374830	-2.508608
9	14	0	2.581280	-2.318057	-1.276218
10	6	0	-4.447602	-0.862153	-2.472240
11	1	0	-4.997282	-0.809937	-3.421966
12	1	0	-4.082659	-1.889394	-2.354215
13	1	0	-5.163542	-0.657641	-1.667604
14	6	0	-2.123019	0.159069	-4.168124
15	1	0	-2.812310	0.363308	-4.998664
16	1	0	-1.264776	0.832638	-4.269948
17	1	0	-1.760454	-0.870331	-4.283589
18	6	0	-3.672769	2.150100	-2.377810
19	1	0	-4.420194	2.340930	-3.159882
20	1	0	-4.149484	2.334184	-1.407019
21	1	0	-2.868441	2.884867	-2.510282
22	6	0	2.284260	-3.884018	-0.232006
23	1	0	1.246071	-4.236197	-0.276944
24	1	0	2.910331	-4.700940	-0.616431
25	1	0	2.554563	-3.728699	0.820449
26	6	0	1.974500	-2.639509	-3.042641
27	1	0	2.119653	-1.753333	-3.673089
28	1	0	2.540075	-3.466811	-3.492601
29	1	0	0.912534	-2.909915	-3.082417
30	6	0	4.448424	-2.011705	-1.349654
31	1	0	4.918544	-2.860926	-1.864992
32	1	0	4.690107	-1.103873	-1.913800
33	1	0	4.908730	-1.919138	-0.360973
34	6	0	-2.694877	0.527158	1.257967
35	6	0	-2.243553	1.271877	2.368020
36	6	0	-4.068136	0.207489	1.204710
37	6	0	-3.126333	1.714732	3.355277
38	1	0	-1.184797	1.509191	2.438566
39	6	0	-4.946819	0.622313	2.207571
40	1	0	-4.439180	-0.387112	0.376279
41	6	0	-4.484956	1.387606	3.283781
42	1	0	-2.751691	2.303326	4.190200
43	1	0	-5.998544	0.352119	2.144519
44	1	0	-5.171656	1.717073	4.059240
45	6	0	3.501676	0.681031	0.318435
46	6	0	4.031156	0.091680	1.485874
47	6	0	4.254876	1.698977	-0.306628

48	6	0	5.265806	0.493832	2.000224
49	1	0	3.462101	-0.687557	1.988440
50	6	0	5.503167	2.074747	0.189908
51	1	0	3.855123	2.176834	-1.198421
52	6	0	6.013541	1.481832	1.351530
53	1	0	5.650445	0.025665	2.903795
54	1	0	6.076344	2.842210	-0.325346
55	1	0	6.979193	1.787334	1.745908
56	17	0	-1.500123	-3.614127	-1.374683
57	8	0	0.147230	2.844685	0.473374
58	6	0	-0.807129	3.917666	0.175858
59	6	0	1.211655	3.338881	1.346194
60	6	0	-0.135670	5.194196	0.677412
61	1	0	-0.987674	3.909535	-0.901287
62	1	0	-1.732800	3.689596	0.714514
63	6	0	0.707315	4.685913	1.858958
64	1	0	1.373709	2.587923	2.122585
65	1	0	2.123023	3.434922	0.749433
66	1	0	-0.868814	5.953226	0.967125
67	1	0	0.508510	5.617223	-0.101703
68	1	0	0.081693	4.553739	2.749890
69	1	0	1.532538	5.356572	2.117650
70	8	0	-0.448506	-2.543542	1.741016
71	6	0	-0.124362	-1.772011	2.941990
72	6	0	-0.857076	-3.903524	2.109696
73	6	0	-0.136862	-2.782734	4.089134
74	1	0	0.845797	-1.296017	2.778256
75	1	0	-0.893597	-1.003539	3.051986
76	6	0	-1.157398	-3.825184	3.602758
77	1	0	-1.705644	-4.171320	1.479724
78	1	0	-0.019683	-4.574785	1.890243
79	1	0	-0.417886	-2.315573	5.038026
80	1	0	0.850878	-3.242273	4.214320
81	1	0	-2.181113	-3.471000	3.771136
82	1	0	-1.045521	-4.797106	4.093283

TS2
298K, 1 atm, gas phase
Thermal correction to Enthalpy = 0.718139 Hartree
Thermal correction to Gibbs Free Energy = 0.580535 Hartree
Sum of electronic and thermal Enthalpies = -2912.009940 Hartree
Sum of electronic and thermal Free Energies = -2912.147544 Hartree
298K, in THF
Sum of electronic and thermal Free Energies= -2912.054464 Hartree

Standard orientation:

Center Number	Atomic Number	Atomic Type	Coordinates (Angstroms) X	Y	Z
1	21	0	-1.519312	0.023678	-0.995532
2	17	0	-2.826119	0.069700	-3.029487
3	6	0	-0.012534	1.621179	-0.733208
4	6	0	-0.374811	1.128588	0.500826
5	6	0	0.374884	-1.128572	0.500803
6	6	0	0.012393	-1.621206	-0.733150
7	21	0	1.519132	-0.023717	-0.995785
8	14	0	-0.356189	-3.305727	-1.487491
9	14	0	0.355945	3.305670	-1.487669
10	6	0	0.706583	-4.634595	-0.638425
11	1	0	0.527894	-5.617988	-1.094014
12	1	0	1.775003	-4.408913	-0.750051
13	1	0	0.493030	-4.715835	0.433787
14	6	0	0.085244	-3.236193	-3.324279
15	1	0	-0.066608	-4.214244	-3.800556
16	1	0	-0.543301	-2.506178	-3.849387
17	1	0	1.128942	-2.938689	-3.481340
18	6	0	-2.187475	-3.830063	-1.379525
19	1	0	-2.315455	-4.819001	-1.841390
20	1	0	-2.560492	-3.901049	-0.350058
21	1	0	-2.829589	-3.132710	-1.932694
22	6	0	2.187292	3.829911	-1.380144
23	1	0	2.829235	3.132489	-1.933429
24	1	0	2.315228	4.818821	-1.842081
25	1	0	2.560553	3.900922	-0.350765
26	6	0	-0.085917	3.236116	-3.324354
27	1	0	-1.129654	2.938614	-3.481166
28	1	0	0.065831	4.214164	-3.800672
29	1	0	0.542500	2.506094	-3.849609
30	6	0	-0.706571	4.634619	-0.638400
31	1	0	-0.527950	5.617986	-1.094078
32	1	0	-1.775029	4.408973	-0.749760
33	1	0	-0.492760	4.715904	0.433756
34	6	0	0.210400	-1.693520	1.853001
35	6	0	1.106310	-1.330756	2.877698
36	6	0	-0.815978	-2.604424	2.175638
37	6	0	1.004813	-1.877575	4.158458
38	1	0	1.880709	-0.601349	2.656948
39	6	0	-0.934368	-3.140179	3.458421
40	1	0	-1.527237	-2.890245	1.406169
41	6	0	-0.017869	-2.783971	4.454866
42	1	0	1.713388	-1.584747	4.929653

43	1	0	-1.736171	-3.840534	3.681811
44	1	0	-0.104600	-3.204840	5.453585
45	6	0	-0.210085	1.693586	1.852973
46	6	0	-1.105815	1.330869	2.877844
47	6	0	0.816358	2.604493	2.175394
48	6	0	-1.004083	1.877732	4.158566
49	1	0	-1.880261	0.601461	2.657261
50	6	0	0.934983	3.140293	3.458136
51	1	0	1.527481	2.890284	1.405788
52	6	0	0.018660	2.784129	4.454759
53	1	0	-1.712523	1.584938	4.929897
54	1	0	1.736831	3.840649	3.681355
55	1	0	0.105574	3.205032	5.453446
56	17	0	2.825624	-0.069813	-3.029943
57	8	0	-3.428341	0.114431	0.204457
58	6	0	-4.509526	-0.858299	0.025378
59	6	0	-3.988413	1.452084	0.393571
60	6	0	-5.801517	-0.039896	-0.006058
61	1	0	-4.322437	-1.402280	-0.903083
62	1	0	-4.463745	-1.543895	0.878305
63	6	0	-5.435270	1.211642	0.810226
64	1	0	-3.373125	1.956718	1.140721
65	1	0	-3.928245	1.986320	-0.562848
66	1	0	-6.646421	-0.594230	0.414087
67	1	0	-6.048097	0.235302	-1.037141
68	1	0	-5.496570	1.009748	1.886463
69	1	0	-6.074188	2.071119	0.585199
70	8	0	3.428340	-0.114422	0.203916
71	6	0	3.988454	-1.452066	0.392965
72	6	0	4.509488	0.858313	0.024646
73	6	0	5.435381	-1.211609	0.809360
74	1	0	3.373300	-1.956688	1.140232
75	1	0	3.928122	-1.986322	-0.563430
76	6	0	5.801475	0.039919	-0.007007
77	1	0	4.322238	1.402290	-0.903784
78	1	0	4.463850	1.543912	0.877576
79	1	0	6.074263	-2.071086	0.584234
80	1	0	5.496870	-1.009701	1.885581
81	1	0	6.047873	-0.235292	-1.038127
82	1	0	6.646447	0.594262	0.412979

IM4

298K, 1 atm, gas phase

Thermal correction to Enthalpy = 0.720152 Hartree

Thermal correction to Gibbs Free Energy = 0.582964 Hartree

Sum of electronic and thermal Enthalpies = -2912.077341 Hartree

Sum of electronic and thermal Free Energies = -2912.214529 Hartree
298K, in THF
Sum of electronic and thermal Free Energies= -2912.247676 Hartree

Standard orientation:

Center Number	Atomic Number	Atomic Type	Coordinates (Angstroms) X	Y	Z
1	21	0	0.004603	-1.784634	-0.122078
2	17	0	-0.097556	-2.610996	-2.499117
3	6	0	-1.485444	-0.375984	-0.648449
4	6	0	-0.740051	0.639857	0.094332
5	6	0	0.693747	0.668293	0.018114
6	6	0	1.398457	-0.342107	-0.773861
7	21	0	-0.120894	0.099085	-2.265740
8	14	0	3.224140	-0.600755	-1.066998
9	14	0	-3.326388	-0.571774	-0.896911
10	6	0	4.304334	-0.293461	0.472687
11	1	0	5.355468	-0.509703	0.235683
12	1	0	4.247693	0.740713	0.828894
13	1	0	4.018064	-0.947382	1.307783
14	6	0	3.920807	0.470956	-2.476645
15	1	0	4.982985	0.245349	-2.644644
16	1	0	3.393279	0.294322	-3.423037
17	1	0	3.841427	1.541412	-2.252562
18	6	0	3.504631	-2.416963	-1.564242
19	1	0	4.563285	-2.580980	-1.807357
20	1	0	3.250057	-3.114509	-0.752953
21	1	0	2.910088	-2.703637	-2.438798
22	6	0	-4.285034	1.063110	-1.021930
23	1	0	-3.873359	1.704868	-1.810989
24	1	0	-5.334489	0.857364	-1.275113
25	1	0	-4.269874	1.635450	-0.088174
26	6	0	-3.637056	-1.524734	-2.511380
27	1	0	-3.108873	-2.484245	-2.543677
28	1	0	-4.711211	-1.725858	-2.623584
29	1	0	-3.324956	-0.950186	-3.394004
30	6	0	-4.123657	-1.601805	0.503038
31	1	0	-5.202543	-1.724335	0.335260
32	1	0	-3.687201	-2.609465	0.556558
33	1	0	-3.996390	-1.122679	1.482260
34	6	0	1.456693	1.730996	0.755871
35	6	0	2.096455	2.760847	0.048127
36	6	0	1.580397	1.710374	2.153410
37	6	0	2.841191	3.736224	0.715641

38	1	0	1.998167	2.803171	-1.033842
39	6	0	2.330007	2.679379	2.825320
40	1	0	1.073609	0.933774	2.722212
41	6	0	2.966127	3.696704	2.107978
42	1	0	3.321302	4.528917	0.146952
43	1	0	2.411675	2.643650	3.909279
44	1	0	3.547443	4.453487	2.628440
45	6	0	-1.456213	1.676523	0.910978
46	6	0	-2.098264	1.323145	2.107700
47	6	0	-1.534734	3.010947	0.483819
48	6	0	-2.792954	2.271859	2.862304
49	1	0	-2.053248	0.290839	2.449313
50	6	0	-2.231685	3.962445	1.232365
51	1	0	-1.053530	3.302710	-0.445858
52	6	0	-2.863388	3.598315	2.425338
53	1	0	-3.281147	1.975553	3.788053
54	1	0	-2.283097	4.989682	0.879932
55	1	0	-3.406579	4.338788	3.006889
56	17	0	-0.211304	1.871526	-3.862582
57	8	0	0.166815	-2.701951	1.897188
58	6	0	-0.926024	-3.275449	2.692555
59	6	0	1.397171	-2.632784	2.699704
60	6	0	-0.249531	-3.845815	3.937151
61	1	0	-1.625510	-2.467668	2.929978
62	1	0	-1.429886	-4.022011	2.073577
63	6	0	0.944289	-2.896107	4.133115
64	1	0	2.076146	-3.407850	2.328686
65	1	0	1.842772	-1.648594	2.539037
66	1	0	0.097099	-4.869120	3.752285
67	1	0	-0.927912	-3.864804	4.795012
68	1	0	1.744175	-3.332889	4.738130
69	1	0	0.623141	-1.964207	4.612413

3a
298K, 1 atm, gas phase
Thermal correction to Enthalpy = 1.030420 Hartree
Thermal correction to Gibbs Free Energy = 0.851151 Hartree
Sum of electronic and thermal Enthalpies = -5359.859848 Hartree
Sum of electronic and thermal Free Energies = -5359.680578 Hartree

Standard orientation:

Center Number	Atomic Number	Atomic Type	Coordinates (Angstroms) X	Y	Z
1	21		0.000038767	-0.000028525	-0.000034431

2	17	-0.000006085	-0.000001792	0.000001936
3	6	-0.000082477	-0.000001339	-0.000006665
4	6	0.000079169	-0.000006407	0.000027379
5	6	-0.000076497	0.000005640	0.000028565
6	6	0.000082343	0.000001735	-0.000006602
7	21	-0.000039258	0.000028489	-0.000035471
8	14	-0.000031794	-0.000013573	0.000011797
9	14	0.000031620	0.000013470	0.000011811
10	6	-0.000006480	-0.000013487	-0.000000052
11	1	0.000010427	-0.000000642	0.000005545
12	1	-0.000002535	0.000010969	-0.000005763
13	1	0.000001840	-0.000001817	-0.000003964
14	6	-0.000004164	-0.000002181	0.000004770
15	1	0.000001568	-0.000001089	0.000001599
16	1	0.000001636	-0.000002409	-0.000003618
17	1	-0.000000063	0.000003577	-0.000000132
18	6	-0.000006259	0.000020250	-0.000017539
19	1	-0.000000448	-0.000002245	0.000005383
20	1	-0.000000963	0.000001312	0.000000421
21	1	0.000002619	0.000000167	0.000000821
22	6	0.000006180	-0.000020493	-0.000017425
23	1	-0.000002579	-0.000000244	0.000000817
24	1	0.000000397	0.000002184	0.000005292
25	1	0.000000961	-0.000001542	0.000000935
26	6	0.000004031	0.000002227	0.000004766
27	1	0.000000082	-0.000003598	-0.000000196
28	1	-0.000001497	0.000001073	0.000001717
29	1	-0.000001625	0.000002387	-0.000003624
30	6	0.000006645	0.000013415	0.000000081
31	1	-0.000010387	0.000000647	0.000005569
32	1	0.000002481	-0.000010948	-0.000005793
33	1	-0.000001820	0.000001844	-0.000003918
34	6	0.000068420	0.000001721	-0.000007443
35	6	0.000001281	0.000011800	-0.000021438
36	6	0.000009154	0.000016377	-0.000032669
37	6	-0.000000458	0.000003544	-0.000001007
38	1	-0.000001527	-0.000004907	0.000004525
39	6	0.000003390	-0.000000432	0.000014872
40	1	-0.000004893	0.000002372	-0.000001653
41	6	-0.000007986	-0.000004505	-0.000004850
42	1	-0.000000815	-0.000000938	0.000001325
43	1	-0.000000498	0.000002026	-0.000000641
44	1	0.000001972	-0.000000696	0.000000164
45	6	-0.000070477	0.000000559	-0.000007961
46	6	-0.000001177	-0.000012220	-0.000020986
47	6	-0.000010034	-0.000016640	-0.000032729

48	6	0.000000679	-0.000003973	-0.000000913
49	1	0.000001645	0.000004164	0.000004335
50	6	-0.000003195	0.000000230	0.000015553
51	1	0.000005413	-0.000002115	-0.000002205
52	6	0.000007945	0.000005210	-0.000005421
53	1	0.000000833	0.000000871	0.000001398
54	1	0.000000502	-0.000002001	-0.000000725
55	1	-0.000001978	0.000000644	0.000000153
56	17	0.000006240	0.000001775	0.000002093
57	8	-0.000003494	-0.000001622	0.000116352
58	6	0.000038711	-0.000005542	-0.000028557
59	6	-0.000007816	0.000046424	-0.000021174
60	6	-0.000019320	0.000007884	-0.000009506
61	1	-0.000005334	-0.000003249	0.000001155
62	1	-0.000000750	-0.000001057	0.000004635
63	6	0.000009410	-0.000008260	-0.000004393
64	1	0.000002687	-0.000008103	-0.000001742
65	1	0.000002752	0.000003765	0.000003427
66	1	0.000001525	0.000001125	0.000002339
67	1	0.000002533	-0.000002522	-0.000001239
68	1	0.000002664	0.000000088	0.000000704
69	1	-0.000000196	0.000000728	-0.000000853
70	8	0.000004352	0.000001915	0.000116703
71	6	0.000007475	-0.000047405	-0.000020883
72	6	-0.000039295	0.000006199	-0.000029536
73	6	-0.000010108	0.000007796	-0.000004515
74	1	-0.000002484	0.000007744	-0.000001434
75	1	-0.000002228	-0.000003858	0.000003546
76	6	0.000019307	-0.000007681	-0.000009454
77	1	0.000005652	0.000003863	0.000001241
78	1	0.000001090	0.000001152	0.000004577
79	1	0.000000066	-0.000000892	-0.000000880
80	1	-0.000003079	-0.000000360	0.000000617
81	1	-0.000002718	0.000002870	-0.000001231
82	1	-0.000001671	-0.000000922	0.000002314

6) References

1. C. Xi, S. Huo, T. H. Afifi, R. Hara and T. Takahashi, *Tetrahedron Lett.*, 1997, **38**, 4099-4102.
2. B. Wei, H. Li, W.-X. Zhang and Z. Xi, *Organometallics,* 2016, **35**, 1458-1463.
3. G. M. Sheldrick, SHELXTL 5.10 for Windows NT: *Structure Determination Software Programs*; Bruker Analytical X-ray Systems, Inc.: Madison, WI, 1997.

4. (a) G. M. Sheldrick, *Acta Crystallogr.*, 2008, **A64,** 112-122. (b) O. V. Dolomanov, L. J. Bourhis, R. J. Gildea, J. A. K. Howard and H. Puschmann, OLEX2: a complete structure solution, refinement and analysis program. *J. Appl. Cryst.*, 2009, **42**, 339-341.
5. A. L. Spek, *Acta Crystallogr.*, 2009, **D65**, 148-155.
6. L. J. Farrugia, *J. Appl. Crystallogr.,* 1997, **30**, 565-565.
7. J. L. Atwood and K. D. Smith, *J. Chem. Soc., Dalton Trans.,* 1974, 921-923.
8. Gaussian 09, Revision C.01: M. J. Frisch, G. W. Trucks, H. B. Schlegel, G. E. Scuseria, M. A. Robb, J. R. Cheeseman, G. Scalmani, V. Barone, B. Mennucci, G. A. Petersson, H. Nakatsuji, M. Caricato, X. Li, H. P. Hratchian, A. F. Izmaylov, J. Bloino, G. Zheng, J. L. Sonnenberg, M. Hada, M. Ehara, K. Toyota, R. Fukuda, J. Hasegawa, M. Ishida, T. Nakajima, Y. Honda, O. Kitao, H. Nakai, T. Vreven, J. A. Montgomery, Jr., J. E. Peralta, F. Ogliaro, M. Bearpark, J. J. Heyd, E. Brothers, K. N. Kudin, V. N. Staroverov, T. Keith, R. Kobayashi, J. Normand, K. Raghavachari, A. Rendell, J. C. Burant, S. S. Iyengar, J. Tomasi, M. Cossi, N. Rega, J. M. Millam, M. Klene, J. E. Knox, J. B. Cross, V. Bakken, C. Adamo, J. Jaramillo, R. Gomperts, R. E. Stratmann, O. Yazyev, A. J. Austin, R. Cammi, C. Pomelli, J. W. Ochterski, R. L. Martin, K. Morokuma, V. G. Zakrzewski, G. A. Voth, P. Salvador, J. J. Dannenberg, S. Dapprich, A. D. Daniels, O. Farkas, J. B. Foresman, J. V. Ortiz, J. Cioslowski and D. J. Fox, Gaussian, Inc., Wallingford CT, 2010.
9. (a) A. D. Becke, *J. Chem. Phys.,* 1993, **98**, 5648-5652; (b) C. Lee, W. Yang and R. G. Parr, *Phys. Rev. B: Condens. Matter Mater. Phys.,*1988, **37**, 785-789.
10. A. E. Reed, L. A. Curtiss and F. Weinhold, *Chem. Rev.*, 1988, **88**, 899-926.
